# GLP-1R–positive neurons in the lateral septum mediate the anorectic and weight-lowering effects of liraglutide in mice

**DOI:** 10.1172/JCI178239

**Published:** 2024-09-03

**Authors:** Zijun Chen, Xiaofei Deng, Cuijie Shi, Haiyang Jing, Yu Tian, Jiafeng Zhong, Gaowei Chen, Yunlong Xu, Yixiao Luo, Yingjie Zhu

**Affiliations:** 1 Shenzhen Key Laboratory of Drug Addiction, Shenzhen Neher Neural Plasticity Laboratory, Shenzhen–Hong Kong Institute of Brain Science, Shenzhen Institute of Advanced Technology, Chinese Academy of Sciences, Shenzhen, China.; 2 Key Laboratory of Molecular Epidemiology of Hunan Province, School of Medicine, Hunan Normal University, Changsha, China.; 3 Department of Neuroscience, City University of Hong Kong, Kowloon Tong, Hong Kong SAR, China.; 4 University of Chinese Academy of Sciences, Beijing, China.; 5 Department of Neonatology, Shenzhen Maternity and Child Healthcare Hospital, The First School of Clinical Medicine, Southern Medical University, Shenzhen, China.; 6 Hunan Province People’s Hospital, The First Affiliated Hospital of Hunan Normal University, Changsha, China.; 7 Faculty of Life and Health Sciences, Shenzhen Institute of Advanced Technology, Chinese Academy of Sciences, Shenzhen, China.; 8 CAS Key Laboratory of Brain Connectome and Manipulation, Brain Cognition and Brain Disease Institute, Shenzhen Institute of Advanced Technology, Chinese Academy of Sciences, Shenzhen, China.

**Keywords:** Metabolism, Neuroscience, Obesity

## Abstract

Liraglutide, a glucagon-like peptide-1 (GLP-1) analog, is approved for obesity treatment, but the specific neuronal sites that contribute to its therapeutic effects remain elusive. Here, we show that GLP-1 receptor–positive (GLP-1R–positive) neurons in the lateral septum (LS^GLP-1R^) play a critical role in mediating the anorectic and weight-loss effects of liraglutide. LS^GLP-1R^ neurons were robustly activated by liraglutide, and chemogenetic activation of these neurons dramatically suppressed feeding. Targeted knockdown of GLP-1 receptors within the LS, but not in the hypothalamus, substantially attenuated liraglutide’s ability to inhibit feeding and lower body weight. The activity of LS^GLP-1R^ neurons rapidly decreased during naturalistic feeding episodes, while synaptic inactivation of LS^GLP-1R^ neurons diminished the anorexic effects triggered by liraglutide. Together, these findings offer critical insights into the functional role of LS^GLP-1R^ neurons in the physiological regulation of energy homeostasis and delineate their instrumental role in mediating the pharmacological efficacy of liraglutide.

## Abstract 2

Liraglutide activates GLP-1 receptor–positive neurons in the lateral septum that are critical for the drug’s anorectic and weight-loss effects and independent of hypothalamic action.

## Graphical abstract

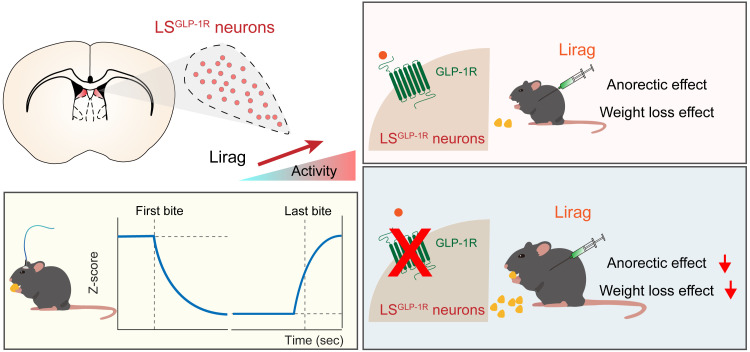

## Introduction

The obesity pandemic continues to pose a global health threat as safe and effective treatments remain elusive. Among the various pharmacological approaches used, glucagon-like peptide-1 (GLP-1) analogs, including liraglutide, are used for clinically marked weight loss (1, 2). As a synthetic GLP-1 analog, liraglutide diminishes appetite and energy intake in both humans and animals, leading to weight reduction (2, 3).

Liraglutide, acting similarly to native GLP-1 (4), modulates energy homeostasis by activating G protein–coupled receptors (2, 5–8). Studies have shown that GLP-1 receptor–expressing (GLP-1R–expressing) cells within the central nervous system (CNS) are pivotal for the anorectic and body weight–reducing effects of peripherally administered liraglutide in rodent models (9–11). The hypothalamus, a central governor of energy balance, has consequently emerged as a key locus for GLP-1R research (12–16). However, genetic deletions of GLP-1Rs in various hypothalamic regions, including the paraventricular nucleus (PVN), arcuate nucleus (Arc), or ventromedial nucleus of the hypothalamus, do not impair the anorectic effects of peripherally administered GLP-1R agonists (17, 18).

Beyond the hypothalamus, attention has also been directed toward the hindbrain region, specifically, the area postrema (AP), which has substantial GLP-1R expression (8, 19–21). Both endogenous GLP-1 and exogenously administered GLP-1 analogs have been found to exert anorectic effects through GLP-1Rs within the AP (22–25). However, some findings, such as the fact that AP lesions do not diminish liraglutide-induced reductions in food intake and body weight (26), raise questions about the precise brain sites where liraglutide’s anorectic effects originate.

Further, GLP-1R is found in the dorsal portion of the lateral septum (LS), a region that projects to key feeding centers in the brain and is believed to regulate gastric emptying after meal consumption (8, 27–29). The observed anorectic effects following local microinfusion of GLP-1R agonists into the LS suggest a role of GLP-1Rs in this region in reducing food intake (30, 31). Yet the physiological functions of GLP-1R–positive neurons in the LS (LS^GLP-1R^ neurons), and whether these neurons are necessary for liraglutide’s anorectic effects, remain largely unknown.

To unravel the above questions, we investigated the physiological and pharmacological roles of GLP-1Rs in the LS. Combining CRISPR/Cas9–mediated targeted knockdown of receptors in a specific group of neurons with neural circuit manipulation techniques, we dissected the function of LS GLP-1 receptors and LS^GLP-1R^ neurons and uncovered an essential role for these neurons in physiological satiation, as well as a role in generating liraglutide-induced anorectic and weight-reducing effects. Our findings delineate the central neural action of the widely used anti-obesity drug liraglutide and provide new insights into the homeostatic regulation of energy and body weight.

## Results

### GLP-1R–expressing neurons in the LS are activated by liraglutide.

We initiated our examination by performing immunostaining to define the distribution of endogenous GLP-1Rs within the mouse brain (Supplemental Figure 1A; supplemental material available online with this article; https://doi.org/10.1172/JCI178239DS1). The specificity of the anti–GLP-1R antibodies was validated in wild-type and GLP-1R–knockout mice (Supplemental Figure 1, B and C). We found densely clustered GLP-1R–positive neurons prominently located in the dorsal portion of the LS (dLS) (Supplemental Figure 1A), a region implicated in energy control according to previous work from our group and other groups (32, 33). A large number of GLP-1R–positive cells were also detected throughout various hypothalamic nuclei, mainly in the PVN, dorsomedial hypothalamic nucleus (DMH), and Arc, and among scattered neurons in the lateral hypothalamus. A considerable GLP-1R–positive presence was also noted in the hindbrain, including the AP and the external cuneate nucleus. Other brain regions that exhibited considerable GLP-1R expression included the ventral pallidum, anterior pretectal nucleus, pontine nuclei, dentate gyrus, and lateral reticular nucleus (Supplemental Figure 1A).

**Figure 1:**
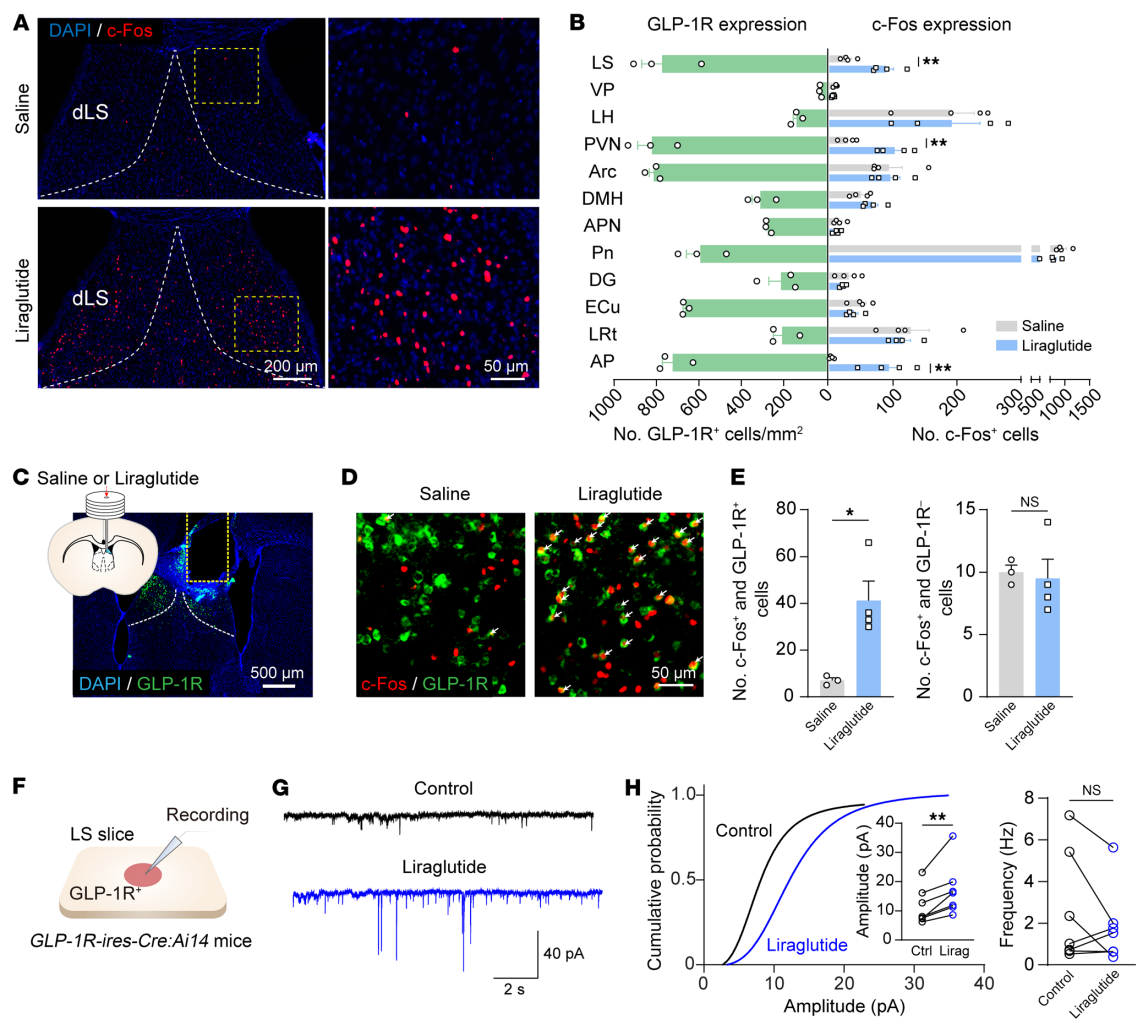
GLP-1R–expressing neurons in the LS are activated by liraglutide. (**A**) Representative image showing c-Fos expression in the dorsal lateral septum (dLS) following i.p. injection of either saline or liraglutide. Scale bars: left, 200 μm; right, 50 μm. (**B**) Left: Quantification of GLP-1R^+^ neuron densities across various brain regions. Right: Quantification of c-Fos^+^ neurons in corresponding brain regions after i.p. injection of saline or liraglutide. VP, ventral pallidum; LH, lateral hypothalamic area; PVN, paraventricular hypothalamic nucleus; Arc, arcuate hypothalamic nucleus; DMH, dorsomedial hypothalamic nucleus; APN, anterior pretectal nucleus; Pn, pontine nuclei; DG, dentate gyrus; ECu, external cuneate nucleus; LRt, lateral reticular nucleus; AP, area postrema. (**C**) Representative image illustrating position of infusion cannula above GLP-1R–positive neurons. Scale bar: 500 μm. (**D**) Representative image showing c-Fos expression in LS^GLP-1R^ neurons. Scale bar: 50 μm. (**E**) Quantification of c-Fos^+^ GLP-1R^+^ (left) and c-Fos^+^ GLP-1R^–^ cells (right) after administration of saline or liraglutide. Unpaired, 2-tailed *t* test; left: *t*_(5)_ = 3.454, *P* = 0.0182; right: *t*_(5)_ = 0.2629, *P* = 0.8031. Means ± SEM. (**F**) Schematic of whole-cell recording from tdTomato–positive neurons in *GLP-1R-ires-Cre:Ai14* slices. (**G**) Representative trace of spontaneous excitatory postsynaptic currents in LS^GLP-1R^ neurons before and after liraglutide application. (**H**) Left: Cumulative amplitude probability plots. Inset and right: Pooled data; *n* = 7 cells from 2 animals; paired 2-tailed *t* test; *t*_(6)_ = 4.017/1.256, *P* = 0.0070/0.2557. *P < 0.05, **P < 0.01.

If a brain region mediates the anorectic effect of GLP-1R agonists, its activity should be modulated by liraglutide. Thus, we systematically mapped brain activation induced by intraperitoneal (i.p.) administration of liraglutide with immunohistochemistry for c-Fos, an early marker of neuronal activity. Liraglutide induced a substantial elevation of c-Fos expression compared with saline controls in the LS, the PVN, and the AP, whereas there was no elevation in other GLP-1R–rich regions (Figure 1, A and B, and Supplemental Figure 1D). As previously noted, ablating GLP-1Rs in the PVN or the AP does not diminish the anorectic or weight-lowering effects of liraglutide (17, 26). This led us to ask whether GLP-1Rs in the LS contribute to the pharmacological effects of liraglutide.

Then we labeled GLP-1R–positive neurons in the LS by crossing *GLP-1R-ires-Cre* mice with *Ai14* reporter mice. Specific expression of red fluorescent protein tdTomato in GLP-1R–positive neurons was observed in the LS (Supplemental Figure 2, A–C). Systematic administration of liraglutide (i.p.) markedly upregulated c-Fos expression in LS^GLP-1R^ neurons (Supplemental Figure 2, D–F), suggesting activation of these neurons. To exclude potential influences of network effects caused by systematic administration, we cannulated above the dLS for localized infusion (Figure 1C). This local infusion of liraglutide into the LS also markedly upregulated c-Fos expression in LS^GLP-1R^ neurons but not in GLP-1R–negative neurons (Figure 1, D and E). Concurrently, a substantial reduction in food intake and body weight was also observed in mice with local liraglutide infusion (Supplemental Figure 2, G–K).

To probe the potential mechanism underlying the activation of LS^GLP-1R^ following liraglutide infusion, we then performed targeted whole-cell patch clamp recording from LS^GLP-1R^ neurons (Figure 1F). Liraglutide dramatically increased the amplitude but not the frequency of excitatory postsynaptic currents in GLP-1–positive neurons. This result is in accordance with a previous report that a GLP-1 analog (exendin-4) enhanced excitatory synaptic transmission through AMPA receptors trafficking in the PVN^CRH^ neurons (16) (Figure 1, G and H).

Together, these findings demonstrate that LS^GLP-1R^ neurons can be effectively activated by liraglutide, possibly through the enhancement of excitatory synaptic transmission.

### GLP-1R knockdown in the LS attenuates the anorectic and body weight–reducing effects of liraglutide.

**Figure 2:**
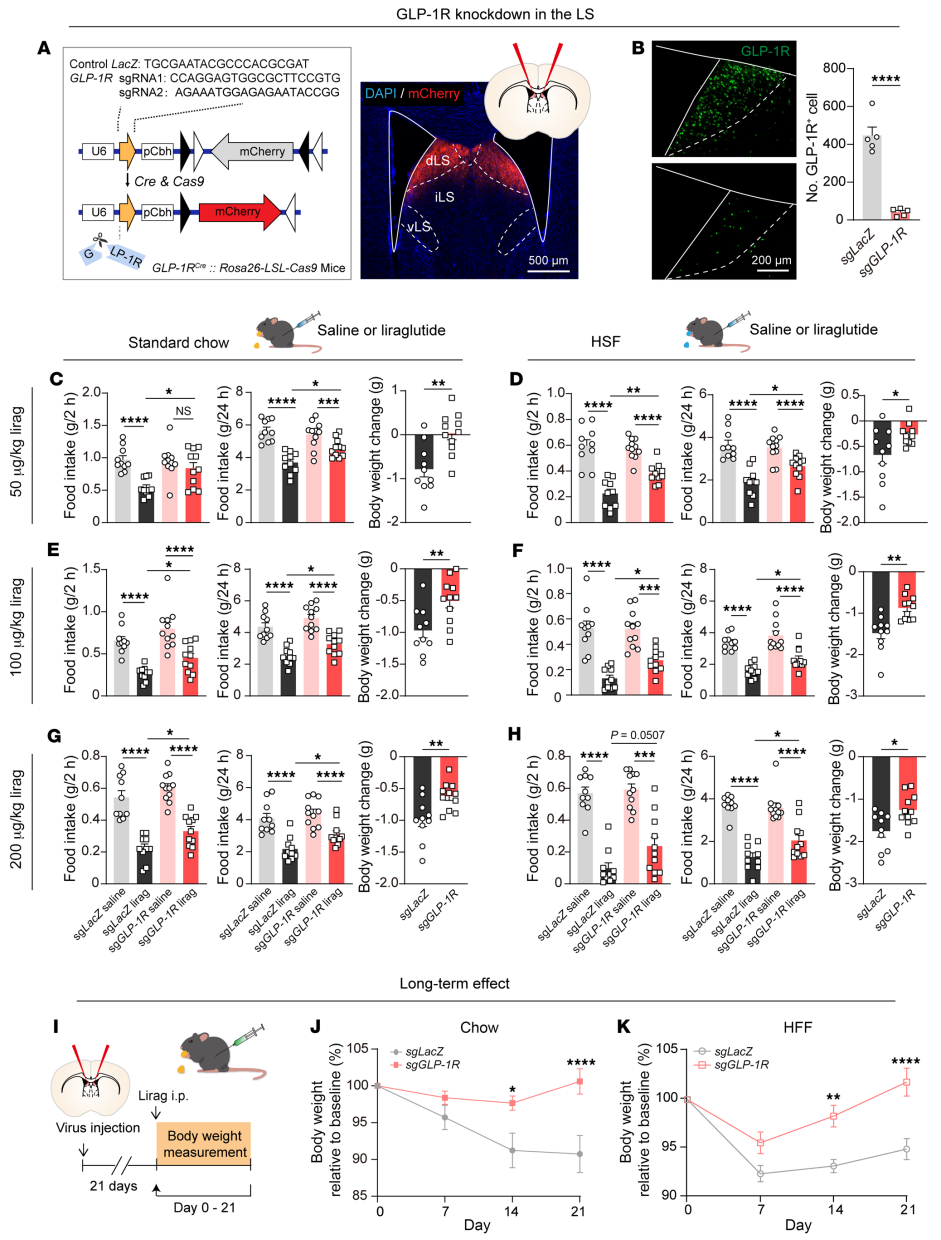
Knockdown of GLP-1Rs in the LS, rather than in the PVN, attenuates the food intake– and body weight–reducing effects of liraglutide. (**A**) Left: Schematic of the viral construct for CRISPR/Cas9–mediated GLP-1R knockdown. Right: Representative image demonstrating virus expression in LS^GLP-1R^ neurons. iLS, intermediate lateral septum; vLS, ventral lateral septum. Scale bar: 500 μm. (**B**) Left: Fluorescence images of dLS depicting GLP-1R immunohistochemistry. Scale bar: 200 μm. Right: Quantification of GLP-1R^+^ neurons in control and GLP-1R knockdown mice (gray: control, *n* = 5; red: GLP-1R knockdown, *n* = 5). Unpaired, 2-tailed *t* test; *t*_(8)_ = 8.952, *P* < 0.0001. Means ± SEM. (**C**–**H**) Attenuation of liraglutide’s anorectic and body weight–reducing effects following GLP-1R knockdown in the dLS on standard chow and a high-sucrose food. Statistical results are provided for varying dosages and durations. Food intake (2 hours, left panels; 24 hours, middle panels): 2-way repeated-measures ANOVA, followed by Šidák’s post hoc test. Body weight (right panels): unpaired, 2-tailed *t* test. Means ± SEM. (**I**) Scheme depicting the investigation of the long-term effect of GLP-1R knockdown in the dLS on liraglutide-induced weight loss. (**J** and **K**) Attenuation of the body weight–reducing effects of chronic systemic liraglutide during a standard chow (**J**) or high-fat food (HFF) diet (**K**) following GLP-1R knockdown in the dLS. Two-way repeated-measures ANOVA; chow: interaction: *F*_(3,39)_ = 5.657, *P* = 0.0026; virus: *F*_(1,13)_ = 10.90, *P* = 0.0057; HFF: interaction: *F*_(3,48)_ = 7.646, *P* = 0.0003; virus: *F*_(1,16)_ = 10.84, *P* = 0.0046. Šidák’s post hoc analysis, *P < 0.05, **P < 0.01, ***P < 0.001, ****P < 0.0001. Means ± SEM.

To elucidate the role of GLP-1Rs in the LS in mediating the anorexigenic effects of liraglutide, we used CRISPR/Cas9 technology to specifically knock down GLP-1R expression in LS^GLP-1R^ neurons. We injected an adeno-associated virus (AAV) containing *GLP-1R*–targeting single-guide RNA (sgRNA) and Cre-inducible mCherry into the dLS of *GLP-1R-ires-Cre* mice bred with Cre-inducible Cas9 knockin mice (34) (Figure 2A). Through this approach, we achieved an approximately 90% reduction in GLP-1R expression in sg*GLP-1R*–targeted neurons compared with controls (Figure 2B).

Remarkably, we found that the knockdown of dLS GLP-1Rs did not influence food intake or body weight in mice at the baseline, regardless of dietary conditions (Supplemental Figure 3, A–E). As expected, acute i.p. injection of various liraglutide dosages markedly reduced food intake in control mice (Figure 2, C–H, and Supplemental Figure 3, F–I). However, the reduction in food intake was dramatically diminished in the GLP-1R–knockdown group (Figure 2, C–H, and Supplemental Figure 3, F–I). Furthermore, GLP-1R knockdown in the LS attenuated body weight reduction induced by liraglutide (Figure 2, C–H, and Supplemental Figure 3, F–I). A prolonged treatment experiment spanning 3 weeks further substantiated these findings. While liraglutide effectively reduced body weight in wild-type mice, the effect was dramatically blunted in mice with GLP-1R knockdown in the LS (Figure 2, I–K).

We also examined the role of GLP-1Rs in other brain regions, such as PVN and Arc, with the same CRISPR/Cas9–mediated knockdown approach. However, neither the targeted knockdown of GLP-1Rs in the PVN nor the Arc altered the anorectic properties following liraglutide (Supplemental Figure 4). Collectively, these findings demonstrate that GLP-1Rs in the LS contribute to the anorectic effects of liraglutide.

### GLP-1R overexpression in the dLS leads to decreased food consumption in satiated mice.

To elucidate the influence of GLP-1R upregulation in LS^GLP-1R^ neurons on food intake, we used a specific AAV vector encoding Cre-inducible mouse GLP-1Rs, complemented with a hemagglutinin (HA) epitope tag, for targeted injection into the dLS of *GLP-1R-ires-Cre* mice (Supplemental Figure 5A). Subsequent immunohistochemical analyses confirmed the overexpression of GLP-1R (Supplemental Figure 5, B and C). This overexpression in the LS led to a substantial decrease in food consumption under sated conditions, but not in fasted mice (Supplemental Figure 5, D–G). The absence of effect in fasted mice may be due to negligible endogenous GLP-1 release in fasted animals (4, 7). Thus, overexpression of GLP-1R in the LS is sufficient to lower food consumption in sated mice.

Next, we assessed the effect of GLP-1R overexpression in the LS on chronic body weight and metabolic parameters. Overexpression of GLP-1Rs did not change chronic body weight compared with that of EYFP-expressing control mice (Supplemental Figure 5H). Overexpression of GLP-1Rs in the LS also did not change oxygen uptake (VO_2_), carbon dioxide discharge (VCO_2_), respiratory exchange ratio, and average energy expenditure (Supplemental Figure 5, I–P).

### Silencing of LS^GLP-1R^ neurons increases food consumption and promotes obesity induced by high-fat food.

Having identified the vital role of LS GLP-1Rs in mediating the anorectic and weight-reducing effects of liraglutide, we sought to elucidate the specific function of LS^GLP-1R^ neurons in the regulation of energy and body weight homeostasis by conducting loss-of-function experiments.

**Figure 3:**
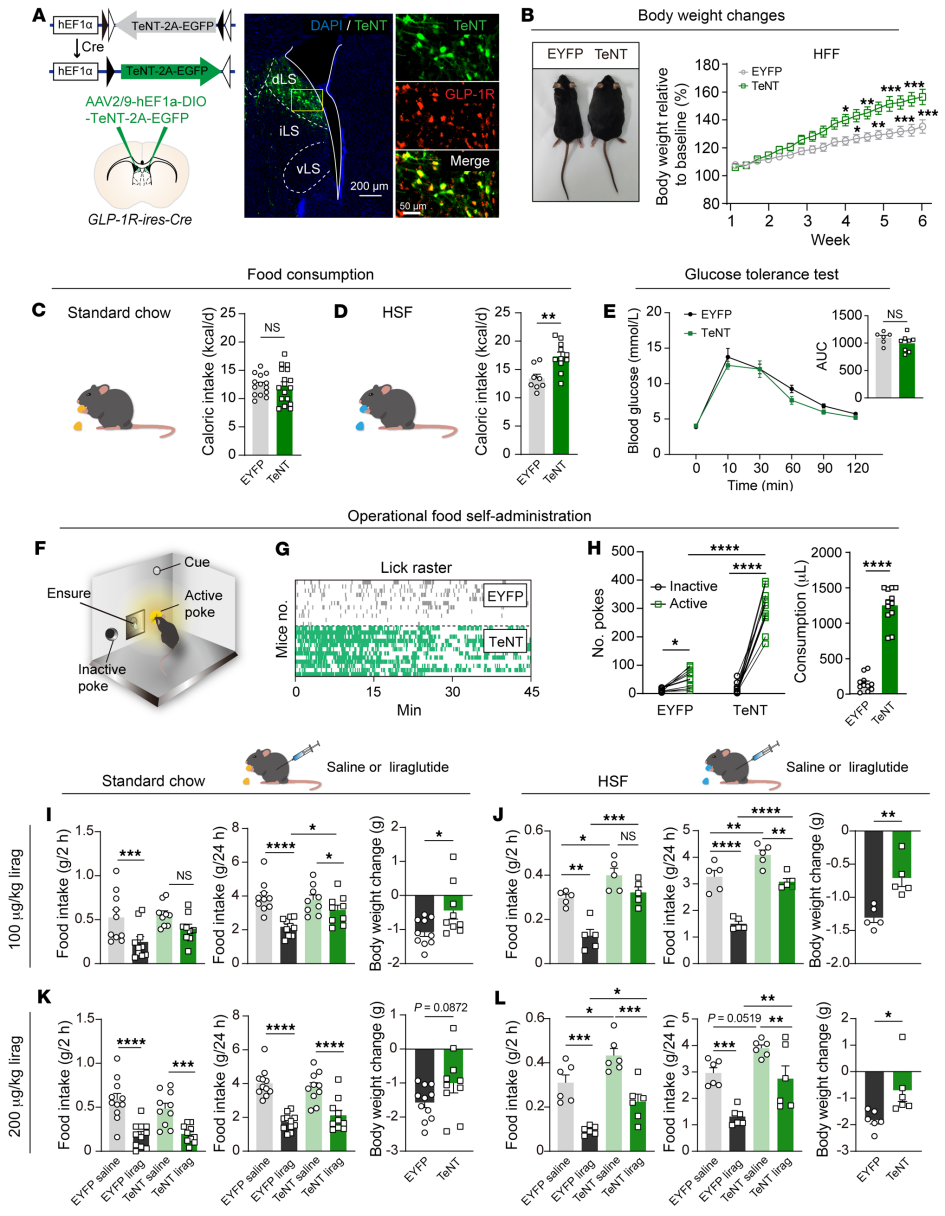
Silencing of LS^GLP-1R^ neurons enhances consumption and reduces liraglutide’s effects on food intake and body weight. (**A**) Representative image showing specific expression of TeNT-2A-EGFP in LS^GLP-1R^ neurons. Scale bars: left, 200 μm; right, 50 μm. (**B**) Left: Images of EYFP- and TeNT-expressing mice after 6 weeks on high-fat diet. Right: Quantification of body weight gain in mice after high-fat diet. Two-way repeated-measures ANOVA; standard chow: *F*_(1,15)_ = 7.203, *P* = 0.0170. Šidák’s post hoc analysis. Means ± SEM. (**C** and **D**) Left: Scheme depicting free consumption of a standard chow and a high-sucrose food paradigm. Right: Quantification of daily solid food intake in EYFP- and TeNT-expressing mice. Unpaired, 2-tailed *t* test; chow: *t*_(29)_ = 0.1347, *P* = 0.8970; high-sucrose food: *t*_(17)_ = 3.529, *P* = 3.529. Means ± SEM. (**E**) Oral glucose tolerance test (oGTT) results for TeNT- and EYFP-expressing mice. Two-way repeated-measures ANOVA; *F*_(5,60)_ = 0.7675, *P* = 0.5769. Inset: Area under the curve (AUC) over 2 hours during oGTT. Unpaired, 2-tailed *t* test; *t*_(12)_ = 1.352, *P* = 0.2014. Means ± SEM. (**F**) Schematic showing Ensure solution self-administration (fixed ratio 1) paradigm. (**G**) Lick patterns for individual mice. (**H**) The TeNT group had a greater number of pokes at the active port and greater Ensure solution consumption than the EYFP group. Two-way repeated-measures ANOVA; *F*_(1,21)_ = 119.2, *P* < 0.0001. Šidák’s post hoc analysis. Unpaired, 2-tailed *t* test; *t*_(21)_ = 13.32, *P* < 0.0001. Means ± SEM. (**I**–**L**) Silencing of LS^GLP-1R^ neurons attenuated the anorectic and body weight–reducing effect of acutely delivered systemic liraglutide during standard chow or high-sucrose diet over varying durations and dosages. Food intake (2 hours, left panels; 24 hours, middle panels): 2-way repeated-measures ANOVA, followed by Šidák’s post hoc test. Body weight (right panels): unpaired, 2-tailed *t* test. Means ± SEM. *P < 0.05, **P < 0.01, ***P < 0.001, ****P < 0.0001.

RNA hybridization has shown that all LS GLP-1R neurons express the vesicular GABA transporter (vGAT), indicative of inhibitory neurons, and none express the excitatory marker vesicular glutamate transporter 2 (vGluT2). Furthermore, among these LS^GLP-1R^ neurons, 39% exhibited somatostatin (Sst) expression, and 23% showed neurotensin (Nts) expression (Supplemental Figure 6, A and B). We then used tetanus neurotoxin (TeNT), a specialized protease known to cleave synaptobrevin-2, thereby blocking neurotransmitter release, as a molecular tool for synaptic silencing (35). To inactivate LS^GLP-1R^ neurons, we injected AAV expressing double-floxed EGFP and TeNT (AAV-DIO-EGFP-2A-TeNT) into the bilateral LS of *GLP-1R-ires-Cre* mice to achieve specific expression of EGFP-2A-TeNT in LS^GLP-1R^ neurons (Figure 3A and Supplemental Figure 6C). On an ad libitum high-fat diet, TeNT mice exhibited greater body weight gain in comparison with EYFP control mice (Figure 3B); however, this difference was not observed under chow-fed condition (Supplemental Figure 7A). Consistent with this, TeNT-mediated silencing of LS^GLP-1R^ neurons markedly increased the consumption of palatable food but not standard chow (Figure 3, C and D).

Considering that several studies have associated the inhibition of central GLP-1R with aggravated glucose tolerance (36), we conducted oral glucose tolerance tests to investigate the role of LS^GLP-1R^ neurons. Interestingly, there was no difference between TeNT and control mice in glucose tolerance (Figure 3E).

We further explored the effect of TeNT expression on liquid food intake during fixed-interval food delivery and operational food self-administration tasks. TeNT expression markedly increased the number of licks and Ensure intake, and the TeNT mice also showed markedly more active poking to obtain Ensure compared with controls (Figure 3, F–H, and Supplemental Figure 7B). Silencing LS^GLP-1R^ neurons, on the other hand, did not increase water consumption (Supplemental Figure 7, C–E), implying that the higher intake of Ensure was not due to the increased need for water.

We also assessed the effects of LS^GLP-1R^ neuronal silencing on metabolic parameters. Both VO_2_ and VCO_2_ were markedly higher in the TeNT mice compared with controls, particularly during the active phase (i.e., the dark cycle) of the day (Supplemental Figure 7, F–I). Despite these differences, the respiratory exchange ratio — the ratio of VCO_2_ produced to VO_2_ consumed — remained unchanged (Supplemental Figure 7, J and K), indicating no substantial differences in the metabolic utilization of carbohydrates and fats between the TeNT mice and the controls. Interestingly, energy expenditure (EE) was also markedly higher in the TeNT mice (Supplemental Figure 7, L and M), possibly because of an increased basal metabolic rate.

Mindful of studies implying an anxiogenic role for the LS (37–39), we explored whether the altered consumption could be attributed to changes in anxiety levels and found that LS^GLP-1R^:TeNT mice exhibited similar anxiety levels and locomotor activity in an open-field test compared with control mice (Supplemental Figure 7, N and O). This suggests that the altered consumption was unlikely to result from changes in anxiety level or locomotion. Together, these data demonstrate that inactivation of LS^GLP-1R^ neurons increases food consumption and promotes obesity induced by high-fat food.

### LS^GLP-1R^ neurons contribute to the anorexigenic and body weight–reducing effect of liraglutide.

To investigate the role of LS^GLP-1R^ neurons in the anorexigenic and weight-reducing effects of liraglutide, we measured the effects of liraglutide on the reduction of food intake and body weight in mice with active (control: AAV-DIO-EYFP) and inactive (TeNT: AAV-DIO-EGFP-2A-TeNT) LS^GLP-1R^ neurons. Liraglutide treatment markedly decreased food consumption and body weight in the control group under both chow-fed and high-sucrose-fed conditions. However, silencing of LS^GLP-1R^ neurons markedly reduced these effects at various doses of liraglutide (Figure 3, I–L, and Supplemental Figure 8).

Nausea is one of the most common adverse side effects reported with GLP-1R agonists, such as liraglutide (40–42). To investigate whether liraglutide-induced nausea requires the action of LS^GLP-1R^ neurons, we used the standard conditioned taste avoidance (CTA) model (40) (Supplemental Figure 7P). Intraperitoneal administration of liraglutide consistently induced a robust CTA across both the TeNT and control groups, with no substantial differences observed between the two (Supplemental Figure 7, Q and R). These findings indicated that the LS^GLP-1R^ neurons were not involved in the liraglutide-induced nausea.

In summary, these results support the notion that LS^GLP-1R^ neurons play an essential role in mediating the anorexigenic and body weight–reducing effects of liraglutide.

### A rapid decrease of LS^GLP-1R^ neuronal activity during the eating episode.

**Figure 4:**
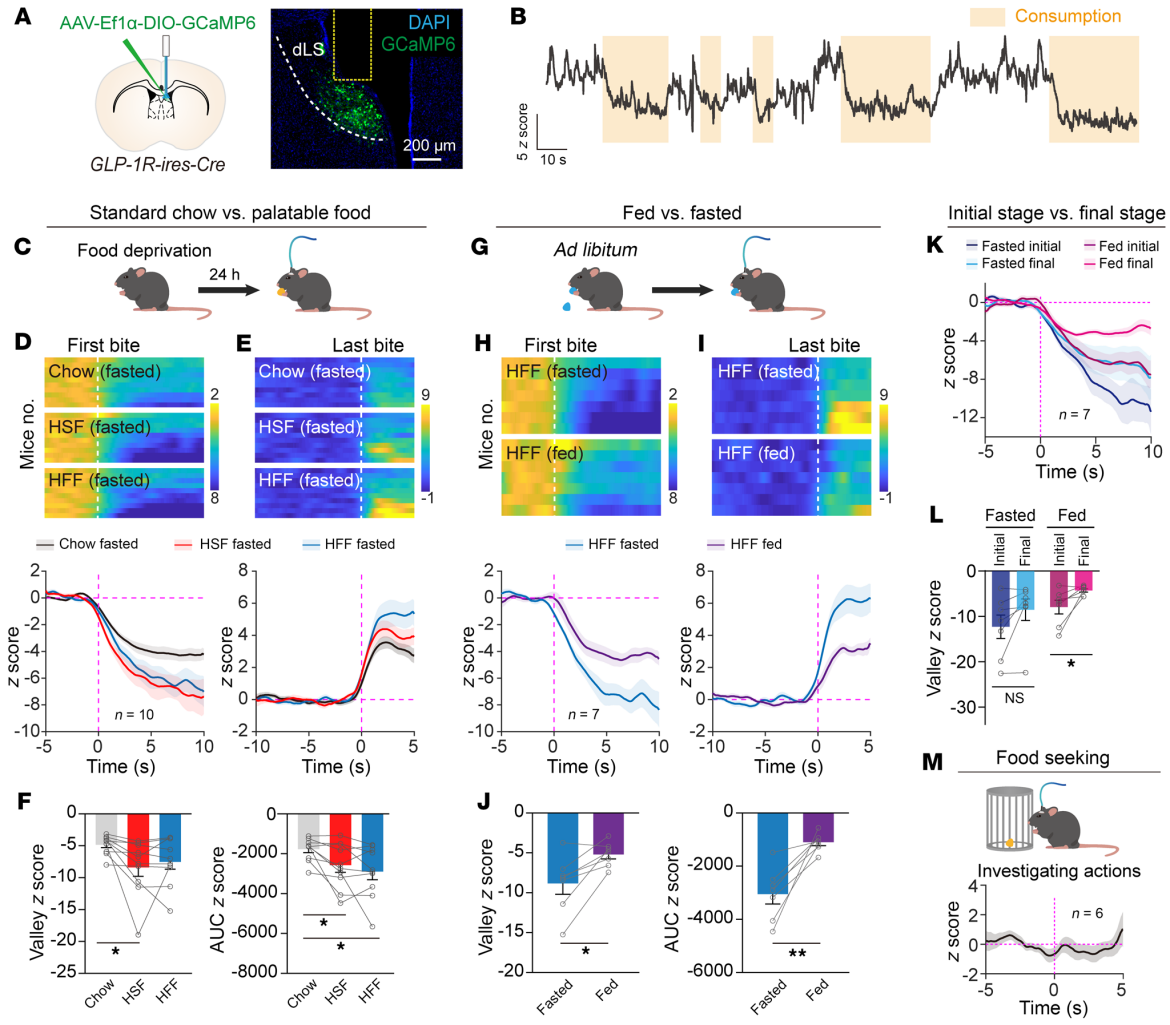
Food consumption rapidly inhibits the activity of dLS^GLP-1R^ neurons, but food seeking has no effect. (**A**) Representative image displaying optic fiber position above GCaMP6-expressing LS^GLP-1R^ neurons. Scale bar: 200 μm. (**B**) Raw trace illustrating real-time Ca^2+^ dynamics within dLS^GLP-1R^ neurons of freely ambulating (moving) mice engaged in feeding. (**C**) Schematic diagram representing the food deprivation protocol used. (**D** and **E**) Comprehensive heatmaps (top) and per-animal stacked plots (bottom) depicting average GCaMP6 responses within dLS^GLP-1R^ neurons, synchronized to the first and last bites during bouts of consumption across various food types (standard chow, high-sucrose, or high-fat) in individually fasted subjects (*n* = 10 animals). (**F**) Calculation of the valley and area under the curve (AUC) for GCaMP6 signals in individual subjects, assessed within a 10-second window immediately after the first bite. (**G**) Schematic illustration of the satiety protocol. (**H** and **I**) Heatmaps (top) and mean traces of GCaMP6 signals (bottom) from dLS^GLP-1R^ neurons, correlating with the commencement (**H**) and cessation (**I**) of high-fat food ingestion in both fasted and sated mice (*n* = 7 animals). (**J**) Quantitative examination of the valley and AUC *z* scores associated with consumption-induced Ca^2+^ responses within dLS^GLP-1R^ neurons across fasted and sated mice. (**K**) Stacked trace of dLS^GLP-1R^-GCaMP6 signals, aligned to the first bite during the initial third and the concluding third of bouts in mice, analyzed under both fasted and fed states (*n* = 7 animals). (**L**) Comparative analysis of the valley of dLS^GLP-1R^-GCaMP6 signals between initial and final feeding stages. (**M**) Stacked plot showcasing the response of dLS^GLP-1R^-GCaMP6 when fasted mice engaged in exploratory actions (*n* = 7 animals). *P < 0.05, **P < 0.01.

Next, to explore the intrinsic activity of LS^GLP-1R^ neurons during naturalistic feeding, we recorded Ca^2+^ signals using fiber photometry under freely moving conditions (Figure 4A). We observed a pronounced decrease in Ca^2+^ signal at the initiation of food consumption in food-deprived mice, and this reduction persisted throughout the eating episode, returning to baseline levels once feeding ceased (Figure 4B and Supplemental Video 1). Interestingly, when mice were exposed to more palatable food sources, such as high-sucrose or high-fat items, rather than standard chow, the decrease in neuronal activity was more pronounced (Figure 4, C–F). This result indicates that the dynamics of LS^GLP-1R^ activity were modulated by food palatability.

To test whether the activity of LS^GLP-1R^ neurons is modulated by energy homeostasis, we presented a pellet of high-fat food to satiated mice. Compared with fasted mice, satiated mice exhibited a markedly smaller inhibitory response during consumption (Figure 4, G–J). To examine this phenomenon more closely, we divided the feeding behavior into 3 stages (initial, middle, and final) and observed a gradually diminishing inhibitory response from the initial to the final stage (Figure 4, K and L). These results demonstrate that the current energy state can actively modulate LS^GLP-1R^ neuronal activity during feeding.

We extended our observation to the food-seeking phase by allowing fasted mice to visually perceive but not physically access a caged food pellet, thus triggering typical seeking behaviors. Despite these actions, no discernible alteration in the Ca^2+^ signal was detected (Figure 4M), demonstrating that food seeking does not modulate dLS^GLP-1R^ neuronal activity.

### Activation of LS^GLP-1R^ neurons reduces food consumption.

**Figure 5:**
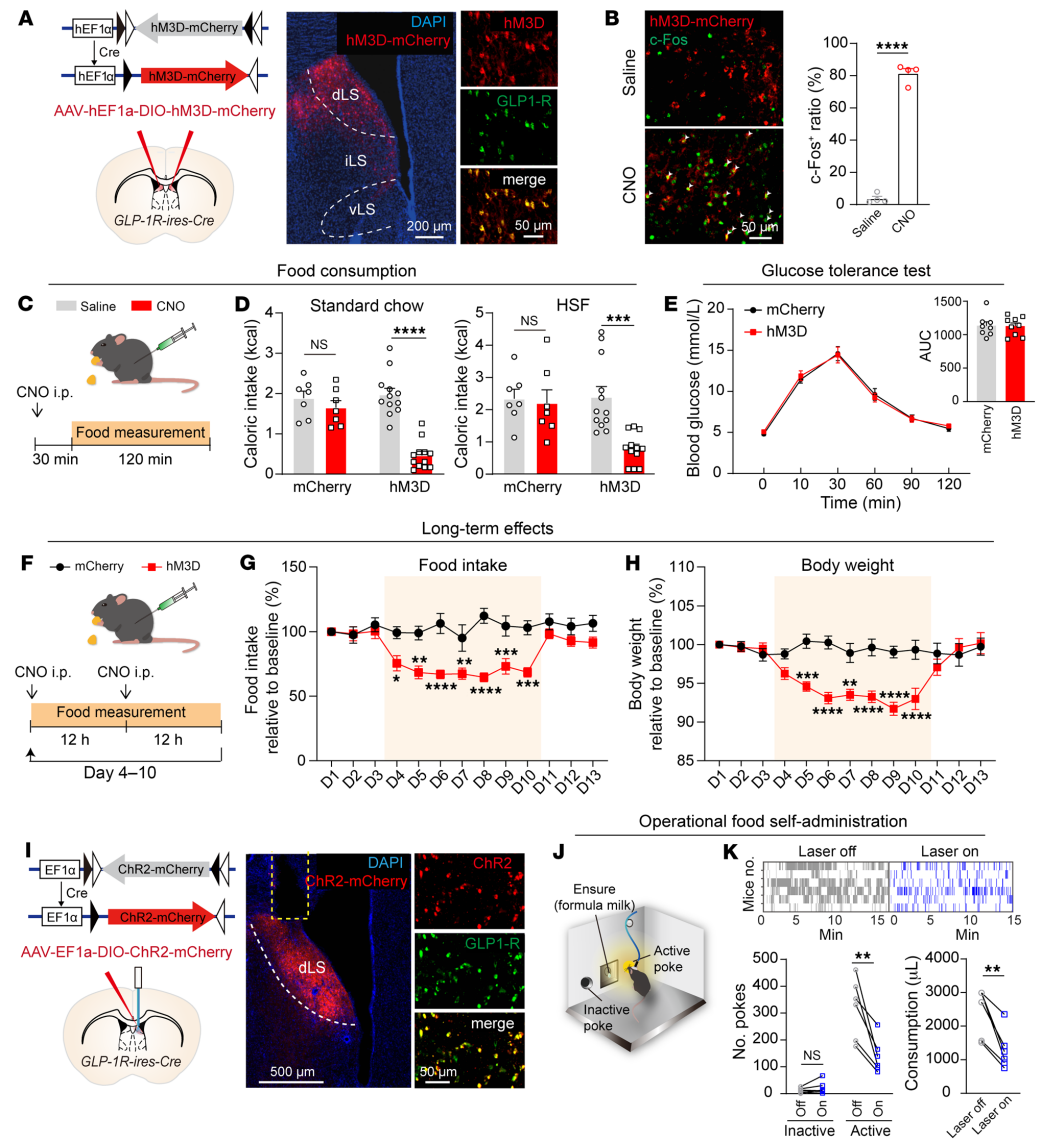
Activation of LS^GLP-1R^ neurons suppresses food consumption and lowers feeding motivation. (**A**) Representative illustration demonstrating specific hM3D-mCherry expression within LS^GLP-1R^ neurons. Scale bars: left, 200 μm; right, 50 μm. (**B**) Left: Representative depiction of robust c-Fos expression. Right: Statistical comparison. Unpaired, 2-tailed *t* test; *t*_(6)_= 23.53, *P* < 0.0001. Means ± SEM. Scale bar: 50 μm. (**C**) Paradigm for analyzing food consumption. (**D**) Effects of CNO injection on reduction of standard chow or high-sucrose food intake specifically in hM3D-expressing mice but not in mCherry-expressing mice. Two-way repeated-measures ANOVA (standard chow, *F*_(1,17)_ = 22.07, *P* = 0.0002; high-sucrose food, *F*_(1,17)_ = 8.784, *P* = 0.0087) followed by Šidák’s post hoc test. Means ± SEM. (**E**) Oral glucose tolerance test (oGTT) results for hM3D- and mCherry-expressing mice. Two-way repeated-measures ANOVA; *F*_(5,75)_ = 0.2521, *P* = 0.9375. Inset: AUC over 2 hours during oGTT. Unpaired, 2-tailed *t* test; *t*_(15)_ = 0.06426, *P* = 0.9496. Means ± SEM. (**F**) Paradigm for analyzing long-term chronic effects of chemogenetic activation of LS^GLP-1R^ neurons on food intake and body weight. (**G**) Prolonged activation of LS^GLP-1R^ neurons suppresses food intake. Two-way repeated-measures ANOVA (*F*_(1,10)_ = 14.19, *P* = 0.0037) followed by Šidák’s post hoc test. Means ± SEM. (**H**) Prolonged activation of LS^GLP-1R^ neurons suppresses body weight. Two-way repeated-measures ANOVA (*F*_(1,10)_ = 8.782, *P* = 0.0142) followed by Šidák’s post hoc test. Means ± SEM. (**I**) Representative image displaying specific ChR2-mCherry expression within LS^GLP-1R^ neurons, accompanied by fiber tracks situated above. Scale bars: left, 500 μm; right, 50 μm. (**J**) Schematic diagram depicting the poke-based Ensure solution intake paradigm. (**K**) Optogenetic stimulation of LS^GLP-1R^ neurons resulted in a decline in the number of pokes at the active port and in consumption of Ensure solution. Left: Two-way repeated-measures ANOVA; *F*_(1,20)_ = 12.27, *P* = 0.0022. Šidák’s post hoc analysis. Right: Paired, 2-tailed *t* test; *t*_(5)_ = 4.932, *P* = 0.0044. **P < 0.01, ***P < 0.001, ****P < 0.0001.

According to our fiber photometry data, we propose that this specific cluster of neurons functions analogously to a brake pad in the regulation of feeding behavior. In the reduced activity state, these neurons facilitate the continuation of feeding. Conversely, when activity is heightened, they play a crucial role in suppressing food intake. To examine whether activation of LS^GLP-1R^ neurons is sufficient to suppress food intake independent of endogenous GLP-1R or exogenous liraglutide, we injected an AAV into the dLS of *GLP-1R-ires-Cre* mice expressing the excitatory Gq-coupled designer receptor exclusively activated by designer drugs (DREADD) hM3D-mCherry in a Cre-dependent fashion (43). This method resulted in the selective expression of hM3D-mCherry in LS^GLP-1R^ neurons (Figure 5A). Intraperitoneal injection of the DREADD agonist clozapine *N*-oxide (CNO) induced robust c-Fos expression in LS^GLP-1R^ neurons transduced with hM3D, indicating increased neuronal activity (Figure 5B).

Next, we performed feeding assays after i.p. injection of CNO or saline. Compared with saline, DREADD-based activation of LS^GLP-1R^ neurons via CNO reduced a 2-hour consumption period of both standard chow and high-sucrose food during the dark period when mice normally consume food actively (Figure 5, C and D). In an overnight fast condition, activation of LS^GLP-1R^ neurons markedly reduced food intake for both standard chow and high-sucrose food (Supplemental Figure 9, A and B). This result suggests that the activity of these neurons can overcome the motivational hunger drive and suppress feeding. Considering prior evidence that GLP-1R activation augments glucose tolerance (44, 45), we probed the potential of LS^GLP-1R^ neurons to modulate glucose homeostasis. Activation of LS^GLP-1R^ neurons did not affect glucose homeostasis (Figure 5E), supporting the role of these neurons in anorectic effects rather than glucose-lowering effects.

We further investigated the long-term effects of chronic LS^GLP-1R^ neuronal activation. CNO was administered bi-daily for 1 week continuously to mice expressing hM3D-mCherry. Chronic CNO administration effectively suppressed both food intake and body weight, which were recovered upon cessation of CNO. In control mice with mCherry expression, the same CNO injection failed to change food intake and body weight (Figure 5, F–H).

To explore whether LS^GLP-1R^ neuronal activity acts as negative or positive valence, we implemented optogenetic activation by expressing channelrhodopsin (ChR2-mCherry) selectively in LS^GLP-1R^ neurons (Figure 5I). In a real-time place preference/avoidance test, LS^GLP-1R^:ChR2 mice exhibited a substantial avoidance of the photostimulation-paired chamber (Supplemental Figure 9, C–E). To examine whether transient activation of LS^GLP-1R^ neurons is sufficient to suppress feeding, we trained LS^GLP-1R^:ChR2 mice to consume Ensure on a fixed ratio 1 schedule, and photostimulation (20 Hz, 1.5 seconds) was synchronized with Ensure administration (Figure 5J). Photostimulation of LS^GLP-1R^ neurons decreased the nose-poking and Ensure consumption (Figure 5K). Collectively, these experiments suggested that LS^GLP-1R^ neurons encoded negative valence and suppressed appetite.

### LS^GLP-1R^ neurons project to multiple brain areas involved in feeding control.

Finally, we sought to elucidate the specific projection targets of LS^GLP-1R^ neurons that are integral to food regulation. We injected AAV-FLEX-tdTomato-T2A-synaptophysin-EGFP into the LS of *GLP-1R-ires-cre* mice (Supplemental Figure 10, A and B). With this tracing strategy, axonal fibers are labeled in red tdTomato, while synapses projecting from LS^GLP-1R^ neurons are labeled in green EGFP (35). We found that LS^GLP-1R^ neurons made major synaptic connections with multiple brain regions (Supplemental Figure 10, C and D), including the nucleus of the horizontal limb of the diagonal band (HDB), the lateral and medial preoptic area (LPO/MPA), the lateroanterior hypothalamic nucleus (LA), the lateral hypothalamic area (LH), the medial tuberal nucleus (MTu), the lateral and medial habenular nucleus (LHb/MHb), the CA3 field of the hippocampus (CA3), and the pyramidal cell layer of the hippocampus (Py). Previous research has suggested that multiple hypothalamic brain areas, including the LH and MTu mentioned above (46, 47), are involved in feeding regulation. Thus, LS^GLP-1R^ neurons project to multiple brain areas that are implicated in feeding regulation.

## Discussion

Liraglutide, a long-acting synthetic GLP-1 analog, suppresses body weight in both humans and preclinical models via a reduction in appetite and energy metabolism (41, 48, 49). Since its approval for obesity treatment in 2014, liraglutide has been widely prescribed worldwide and has generated sales of billions of dollars (50). However, the underlying mechanisms and specific sites of action contributing to its anorectic properties remain obscure. Our findings illustrate that liraglutide activates GLP-1R–expressing neurons in the dorsal LS (dLS) (Figure 1), and knockdown of GLP-1Rs in the dLS, but not the Arc or PVN, diminishes liraglutide’s weight-reduction effects (Figure 2). The silencing of LS^GLP-1R^ neurons enhances food consumption and obesity and diminishes liraglutide’s anorectic effects (Figure 3). During food consumption, there is a decrease in the activity of LS^GLP-1R^ neurons (Figure 4), and chemogenetic or optogenetic activation mimics liraglutide’s anorectic effects (Figure 5). These results emphasize the critical role of LS^GLP-1R^ neurons and LS GLP-1Rs in feeding regulation, not only in orchestrating energy metabolism via endogenous GLP-1 but also in mediating anorectic and body weight–lowering effects of liraglutide.

As is the case with native GLP-1, the anorectic and weight-lowering effects of liraglutide require central GLP-1R activation (4, 9). GLP-1Rs are distributed across the CNS, notably in areas critical for the regulation of food intake and body weight, such as the Arc and the PVN. Other regions that have GLP-1Rs, such as the dLS, have received little attention. Despite prior evidence linking endogenous GLP-1 in the LS with increased satiety (30), the contribution of these GLP-1Rs to the pharmacological effects of liraglutide was unknown. Hence, we employed a sophisticated approach using viral strategies in concert with transgenic animal models to determine the physiological function of GLP-1Rs in the dLS. This allowed us to elucidate with high temporal and spatial precision the essential role these receptors play in the food intake and body weight reduction effects of liraglutide.

Our data reveal that while GLP-1R knockdown in the LS does not alter basal food intake or body weight, TeNT-mediated inactivation of these neurons promotes food intake and weight gain on a high-fat diet. These likely stem from subtle but important methodological differences. Selective knockdown of GLP-1Rs preserves other neuronal functions, allowing continued engagement of LS^GLP-1R^ neurons in neural circuits via alternative receptors and signaling pathways. Conversely, TeNT-mediated synaptic silencing completely blocks neurotransmitter release from LS^GLP-1R^ neurons and shuts down all physiological functions mediated by these neurons (33, 35). Thus, our results suggest that the synaptic activity of LS^GLP-1R^ neurons plays a crucial role in the regulation of food intake and body weight. Upon GLP-1R knockdown, additional signaling pathways beyond GLP-1 are involved to regulate LS^GLP-1R^ neuronal activity and energy homeostasis.

Our data show that GLP-1Rs in the dLS are a crucial contributor of liraglutide’s anorectic and body weight–reducing effects. These receptors likely collaborate with GLP-1Rs in other brain regions, collectively mediating the anorectic and body weight–lowering effects of liraglutide (9, 26, 49). Evidence supporting this concept is provided by the binding of fluorescently labeled liraglutide to neurons in the Arc and specific hypothalamic regions in mice (49). Direct intra-hypothalamic GLP-1R agonism also induces anorexia (15, 17, 51–53). Despite these findings, our data revealed that the knockdown of GLP-1Rs in the Arc and PVN did not alter liraglutide’s anorectic effects. Other studies corroborate this view, showing preserved anorectic effects of peripheral liraglutide after hypothalamic GLP-1R knockdown (17, 18), with no subsequent hypothalamic neuronal activation (17, 54, 55). Thus, while the hypothalamus may play a role, it is likely not the primary locus of action mediating the anorectic effects of GLP-1 and GLP-1 derivatives. The vital contribution of GLP-1Rs in the dLS is clear and should not be overlooked in our understanding of these multifaceted mechanisms.

Liraglutide, along with other GLP-1R agonists, not only diminishes food intake but also frequently elicits some side effects, such as nausea (41, 42, 56). The anorectic and nausea-inducing responses to these agents critically depend on the activation of GLP-1Rs within the central nervous system (CNS) (9–11, 23, 25, 26, 40, 57); whether these effects arise from different brain regions remains an open question. Our data suggest that silencing of LS^GLP-1R^ neurons diminishes the anorectic effect but preserves the nausea response induced by liraglutide (Figure 3 and Supplemental Figure 7, P–R), implying the involvement of other brain regions in nausea. Supporting this, GLP-1Rs in the nucleus tractus solitarius (40), locus caeruleus, and central amygdala have been reported to mediate nausea induced by GLP-1R agonists (52, 58). Thus, our results suggest that targeting GLP-1R in the LS or LS^GLP-1R^ neurons could preserve the anorectic and body weight–lowering effects of liraglutide while avoiding side effects such as nausea.

Our fiber photometry experiments revealed a larger decrease in LS^GLP-1R^ neuronal Ca^2+^ dynamics when mice consumed palatable food as opposed to standard chow. This observation prompted us to hypothesize that alterations in LS^GLP-1R^ neuronal activity exert a more profound influence on palatable food consumption. This potentially explains why the silencing of LS^GLP-1R^ neurons impacted food intake and body weight only when mice were exposed to palatable food, leaving the effects on standard chow unaltered. In the context of Ensure, identified as highly palatable, more conspicuous phenotypes were observed, resonating with the above findings.

Collectively, these data not only underscore the complexity of the underlying mechanisms but also demonstrate the integral involvement of LS^GLP-1R^ neurons in mediating the effect that liraglutide has on food intake and body weight. Such insights reaffirm the importance of GLP-1Rs in the dLS and pave the way for targeted interventions in the future.

## Methods

### Sex as a biological variable

Our study examined male and female animals, and similar findings are reported for both sexes.

### Animals

All animal husbandry and experimental procedures were approved by the Animal Care and Use Committees at the Shenzhen Institute of Advanced Technology, Chinese Academy of Sciences (CAS). Adult male C57BL/6J mice (3–5 months old; Guangdong Medical Laboratory Animal Center), *GLP-1R-ires-Cre* mice (Shanghai Model Organisms, NM-KI-200134), GLP-1R-KO mice (Shanghai Model Organisms, NM-KO-190803), *Ai14:Rosa26-loxP-STOP-loxP-tdTomato* mice (The Jackson Laboratory, 007908), and *Rosa26-LSL-Cas9* mice (The Jackson Laboratory, 024857) were used for this study. Mice were maintained at a constant temperature of 22°–25°C, following a 12-hour light/12-hour dark cycle.

### Stereotaxic surgery

#### Virus injections.

Mice were anesthetized using pentobarbital (80 mg/kg). Stereotaxic injections delivered 200–300 nL of the respective virus into the dLS (coordinates: anteroposterior [AP], +0.75 mm; mediolateral [ML], +/– 0.30 mm; dorsoventral [DV], –2.30 mm), the Arc (coordinates: AP, +1.94 mm; ML, +/– 0.30 mm; DV, –5.75 mm), and the PVN (coordinates: AP, +0.75 mm; ML, +/– 0.25 mm; DV, –4.50 mm) at a rate of 60 nL/min. After injection, mice were given a minimum recovery period of 3 weeks before initiating behavioral experiments. All the virus information is summarized in Supplemental Table 1.

#### Optic fiber and infusion cannula implantation.

Twenty minutes after the unilateral injection of AAV2/9-hEF1a-DIO-hChR2(H134R)-mCherry-WPRE-pA into the dLS, optic fibers (200 μm diameter; numerical aperture 0.37) were implanted. These fibers were positioned above the right dLS with the following coordinates: 0.75 mm anterior, 0.30 mm lateral, and 2.10 mm ventral from the dura. The ferrule was then securely attached to the skull using light-curable resin. After implantation, the incision was sutured, and antibiotic ointment was applied to the surgical site. Optogenetic and fiber photometry studies commenced after a 3-week post-surgery recovery period.

Infusion cannulas were implanted over the unilateral LS (coordinates: AP, +0.75 mm; ML, –0.30 mm; DV, –2.10 mm) and were anchored to the skull using dental cement. After the procedure, a dummy cannula was inserted, topped with a cap, ensuring that the guide cannula remained unobstructed. A recovery period of a minimum of 2 weeks was provided to the mice before initiation of behavioral tests.

At the study’s completion, the locations of virus expression and optic fiber placement were verified post hoc in all animals, and this information is summarized in Supplemental Figure 11.

### Fiber photometry

Fiber photometry recordings were initiated at least 3 weeks after surgery using the fiber photometry system ThinkerTech. A 470 nm blue LED light (30 μW) was channeled through a singular implanted ferrule. The resulting signals were analyzed using a custom MATLAB script. The *z* score was determined as (*x* − μ)/σ, where the mean and standard deviation were derived from the signal 5 seconds before stimulation.

For recordings during free-moving feeding, mice — either food-deprived for 24 hours or fed normally — were introduced to a transparent acrylic enclosure measuring 15 × 15 × 30 cm and then connected to the fiber photometry apparatus. A 5-minute habituation period was followed by the gentle placement of a food pellet (choices included chow or high-sucrose or high-fat content) in the center of the box. The system simultaneously captured the free-feeding behavior and the GLP-1R:GCaMP signals for 15 minutes. All obtained data were segmented and time-aligned according to individual behavioral bout onsets. Fluorescence signals were normalized using *z* scores, taking into account the mean and standard deviation from time windows of −5 to 0 seconds before the first bite and −10 to 0 seconds leading up to the last bite. Any behavioral bouts lasting fewer than 10 seconds were disregarded.

### Behavioral assays

#### Drug administration.

Clozapine *N*-oxide (CNO; Enzo Life Sciences, BML-NS105) was injected i.p. at 2 mg/kg. This was a within-subjects design, and all mice were treated with both CNO and saline vehicles in a counterbalanced approach. Liraglutide (GL Biochem) was administered i.p. 30 minutes before dark onset at doses of 200, 100, or 50 μg/kg. During our study on the effects of LS^GLP-1R^ neuron inactivation and lateral septal GLP-1R knockdown, both test and control groups were given drugs and vehicles in a counterbalanced method in a between-subjects design.

#### Solid food intake assays.

Mice were individually housed for a minimum of 3 days before assay. Daily food (standard chow or high-sucrose) allocations of about 20 g were provided, and cages were refreshed daily. Food consumption was manually determined early in the dark phase (8–10 pm), with caloric intake deduced from a burden sheet. Diet shifts were introduced according to experimental needs, and each shift was given a 3-day acclimation period before intake measurements. For liraglutide treatment, intake was measured 30 minutes after administration of liraglutide. In chemogenetic activation experiments to study acute activation effects, CNO was administered i.p. 30 minutes before the dark phase, and food intake during the 2 hours prior to the dark phase was measured. To study chronic long-term effects, CNO was administered i.p. every 12 hours for a continuous period of 7 days, with daily measurements of food intake and body weight. All the food ingredients are summarized in Supplemental Table 2.

#### Liquid food intake assays.

Before experiments, mice were individually housed with unrestricted access to food and water. For the duration of the study, mice were placed in an operant conditioning chamber outfitted with a nose-poke system (22 × 16 × 15 cm; AniLab). The time stamps of licks and nose pokes were collected, and subsequent data analysis was performed using a custom MATLAB script.

During the fixed-interval delivery assay, a 10 μL droplet of Ensure solution (Abbott) was offered every 10 seconds, totaling 180 presentations. Mice underwent training to lick the spout from day 1 to day 3. Data collection commenced on day 4.

During the self-administration food intake (fixed ratio 1) assay, one port was randomly selected as the “active” port. Poking this active port yielded an Ensure solution reward, while pokes to inactive ports went unrewarded. The test spanned 3 consecutive 45-minute daily sessions, with data from the third day being the focus of analysis. When this test was paired with optogenetic manipulation experiments, mice received intracranial stimulation (473 mm, 10–15 mW, 20 Hz, 1.5 seconds) concurrent with the nose poke on day 4, and data were subsequently captured.

#### Real-time place preference/avoidance.

In the real-time place preference/avoidance (RTPP/A) assay, a specially designed 2-chamber setup was used. Before testing, mice were carefully connected to the optical fiber. On the first day, mice roamed freely within the apparatus for 15 minutes, in the absence of any light stimulation. The following day, one side of the apparatus, chosen in a counterbalanced manner, was designated for stimulation. Upon entry to this side, the mouse received a 20 Hz laser pulse (473 nm, with a 20-millisecond pulse duration and intensity of 5–10 mW/mm^2^ per side). This stimulation ceased once the mouse moved back to the opposite chamber. Place preference scores were computed by measuring the time spent in the stimulated chamber and then contrasting this with the baseline duration.

#### Open-field test.

For behavioral testing, mice were moved to the testing environment 3 hours before the test, where they acclimated to the new surroundings for the duration. Before each test, the equipment was meticulously cleaned using a 20% ethanol solution to ensure the removal of residual odors from previous test mice. Mice were then introduced to a square open-field arena crafted from plastic, measuring 40 cm by 40 cm. Their movements within this space were continuously tracked from above by a camera and using custom MATLAB code, operating at a 30 Hz capture rate. For analysis, the arena was conceptually bisected into 2 distinct zones: a central square of 20 cm by 20 cm, and the surrounding peripheral space. Both the overall movement patterns and the proportion of time the mice allocated to the central region were subjects of our analytical focus.

#### Conditioned taste avoidance.

The experimental procedure was adapted from a previous report (40). In brief, mice were individually housed and deprived of water for 24 hours on day 1. Then, for 3 consecutive days, the mice were simultaneously presented with 2 water bottles for 90 minutes at 6 pm, followed by an i.p. injection of saline. On day 5, each mouse was introduced to 2 bottles of a new beverage (0.2% saccharin mixed with 0.15% cherry-flavored or grape-flavored Kool-Aid) during the regular water presentation time, followed by an i.p. injection of saline. On day 6, the procedure was repeated but with a different Kool-Aid flavor (if cherry was used on day 5, grape was used on day 6, and vice versa), followed by an i.p. injection of liraglutide (200 μg/kg). Days 7 and 8 replicated the procedures of days 5 and 6, respectively. To counteract potential taste preference bias, the order of cherry-flavored and grape-flavored beverages was balanced within each group. Finally, on day 9, mice were presented with both flavors simultaneously, with the water bottles being switched every 30 minutes. The total liquid consumption from both bottles was recorded over a 90-minute period.

### Body weight measurement

Body weight was consistently measured manually at the same daily interval. In experiments involving TeNT-induced synaptic inactivation and GLP-1R overexpression, mice were provided with either standard chow or high-fat food. For experiments focused on the knockdown of LS GLP-1Rs, mice received daily i.p. injections of liraglutide at a dose of 200 μg/kg of body weight. For chemogenetic activation experiments, mice received semidiurnal i.p. injection of CNO at a dose of 2 mg/kg body weight.

### Energy expenditure measurement

Mice were individually housed and acclimated to metabolic cages (TOW-INT Tech.) for at least 3 days before testing under a 12-hour light/12-hour dark cycle. Energy expenditure, VO_2_, VCO_2_, respiratory exchange ratio, food intake, and water intake were measured continuously and simultaneously at a sampling frequency of 5 times per minute. Data were exported to Excel and analyzed using MATLAB (MathWorks). Light and dark cycles were indicated by white (6 am to 6 pm) and black (6 pm to 6 am), respectively. Food and water were freely available during testing. The gas sensors for CO_2_ and O_2_ were calibrated before each test.

### Oral glucose tolerance test

Blood glucose was measured by making a small horizontal incision on the mouse’s tail end using a razor blade. A drop of blood was gently massaged out, and its glucose concentration was determined using a Bayer blood glucose meter. Throughout this procedure, food was withheld. In the TeNT-induced synaptic inactivation experiments, blood glucose was gauged after an overnight fast of 16 hours. Subsequently, these fasting mice were given an oral dose of 1.5 g/kg 30% d-glucose solution (Sigma-Aldrich, catalog G6125). Blood samples were taken at baseline, and then at intervals of 10, 30, 60, 90, and 120 minutes after glucose administration. For chemogenetic activation experiments, fasting blood glucose levels (the baseline at time zero) were measured 30 minutes after administration of CNO injections.

### Histological procedures

Mice were euthanized using a pentobarbital sodium overdose and then transcardially perfused with phosphate-buffered saline (PBS; pH 7.4), followed by 4% paraformaldehyde (PFA). The brains underwent postfixation in 4% PFA for 8–12 hours and were subsequently dehydrated in 30% sucrose for 24–48 hours or until fully submerged. The tissue was then embedded in Tissue-Tek OCT compound (Sakura) and frozen with dry ice. Using a cryostat (Leica), 40-μm sections were prepared, with free-floating sections stored in PBS.

For staining, sections were initially rinsed with PBS 3 times, 10 minutes each. They were then blocked at room temperature using a solution of 10% normal goat serum and 0.3% Triton X-100, followed by an overnight incubation at 4°C with the primary antibody. After three 10-minute washes with PBST, sections were incubated with a fluorophore-linked secondary antibody for 2 hours and then counterstained with DAPI (1:3,000 dilution). Images were captured using an Olympus Virtual Slide Microscope (VS120-S6-W) and later analyzed by an evaluator unaware of the experimental group identities. Antibody information is summarized in Supplemental Table 3.

### In situ hybridization

Mouse brains were sectioned into 18-μm slices using a Leica cryostat and affixed to SuperFrost Plus microscope slides. Probes targeting GLP-1R (catalog 418851-C3), vGAT (catalog 319191-C3), vGluT2 (catalog 319171), somatostatin (Sst; catalog 404631), and neurotensin (Nts; catalog 420441) were specifically designed and validated by Advanced Cell Diagnostics. All fluorescence in situ hybridization experiments were performed using the RNAscope v2 Assay (catalog 323100) following the manufacturer’s guidelines. Briefly, brain slices were air-dried at 39°C for 2 hours, washed with 1× PBS, treated with 3% hydrogen peroxide in methanol and Target Retrieval buffer (Advanced Cell Diagnostics [ACD]) for 15 minutes, and subjected to RNAscope Proteinase III incubation at 40°C for 15 minutes. The brain slices were subsequently incubated with mRNA probes at 40°C for 2 hours. Specific signals were then amplified using a multiplexing amplification solution, and detection was carried out using the TSA Plus Fluorescence Kit (Advanced Cell Diagnostics, 322809). Subsequent quantitative analyses were performed using ImageJ software (NIH).

### Cell counting

For cell counting of c-Fos^+^, tdTomato^+^, and GLP-1R^+^ markers, we obtained 40 μm coronal sections from the target brain areas of each mouse. Images were captured using an Olympus Virtual Slide Microscope (VS120-S6-W). A custom MATLAB script was used for cell counting, while colocalization was visually determined.

### Electrophysiological recordings

Coronal 250- to 300-μm slices containing the LS were prepared using a vibratome (Leica, VT-1000S) in an ice-cold choline-based solution containing (in mM) 110 choline chloride, 2.5 KCl, 0.5 CaCl_2_, 7 MgCl_2_, 1.3 NaH_2_PO_4_, 1.3 Na-ascorbate, 0.6 Na-pyruvate, 25 glucose, and 25 NaHCO_3_, saturated with 95% O_2_ and 5% CO_2_. Slices were incubated in 32°C oxygenated artificial cerebrospinal fluid (in mM: 125 NaCl, 2.5 KCl, 2 CaCl_2_, 1.3 MgCl_2_, 1.3NaH_2_PO_4_, 1.3 Na-ascorbate, 0.6 Na-pyruvate, 25 glucose, and 25 NaHCO_3_) for at least 1 hour before recording. Slices were transferred to a recording chamber and superfused with 2 mL/min artificial cerebrospinal fluid. Patch pipettes (2–5 MΩ) pulled from borosilicate glass (World Precision Instruments, PG10150-4) were filled with a Cs-based low-Cl^–^ internal solution containing (in mM) 135 CsMeSO_3_, 10 HEPES, 1 EGTA, 3.3 QX-314, 4 Mg-ATP, 0.3 Na-GTP, 8 Na_2_-phosphocreatine, 290 mOsm/kg, adjusted to pH 7.3 with CsOH. The whole-cell voltage-clamp recording was performed at room temperature with a Multiclamp 700B amplifier and a Digidata 1440A (Molecular Devices). Data were sampled at 10 kHz and analyzed with Clampfit (Molecular Devices) or MATLAB. The excitatory postsynaptic currents were recorded at a holding potential of –70 mV.

### Statistics

All experiments were conducted blinded. All statistical data can be found in the figure legends. Statistical significance was set at *P < 0.05, **P < 0.01, ***P < 0.001, ****P < 0.0001. Data are presented as means ± SEM. Sample sizes and sex distribution for figures are listed in Supplemental Table 4.

### Study approval

All procedures performed were approved by the Animal Care and Use Committees at the Shenzhen Institute of Advanced Technology, Chinese Academy of Sciences (CAS).

### Data availability

All data sets generated or analyzed during this study are included in the published article and its supplemental material. The supporting data values are available in the Supporting Data Values file. Any additional information required to reanalyze the data reported in this paper is available upon request.

## Author contributions

ZC, XD, CS, YT, JZ, GC, HJ, and YX performed the experiments. YL and YZ supervised the analysis. ZC and YZ designed all the experiments and wrote the manuscript with input from the coauthors.

## Supplementary Material

Supplemental data

Supplemental video 1

Supporting data values

## Version 1 09/03/2024

Electronic publication
