# Supplementary material for: GLP-1R–positive neurons in the lateral septum mediate the anorectic and weight-lowering effects of liraglutide in mice
Source: J Clin Invest. 2024 Sep 3;134(17):e178239. doi: 10.1172/JCI178239 (PMC11364389; doi:10.1172/JCI178239)
Supplement: Supplemental data [file jci-134-178239-s041.pdf]

Supplemental Table and Figures

Supplemental Table 1. The information of virus strains.

Virus strains	Source	Identifier
AAV2/9-hEF1a-DIO-GCaMP6s-WPRE-pA	Taitool Bioscience	S0351-9
AAV2/9-hSyn-DIO-hM3D(Gq)-mCherry-WPRE-pA	Taitool Bioscience	S0192-9
AAV2/9-hEF1a-DIO-hChR2(H134R)-mCherry-WPRE-pA	Taitool Bioscience	S0170-9
AAV2/9-CAG-DIO-EGFP-2A-TetTox-pA	Taitool Bioscience	S0235-9
AAV2/9-hEF1a-DIO-EYFP-WPRE-PA	Taitool Bioscience	S0196-9
AAV2/9-hEF1a-DIO-mCherry-WPRE-PA	Taitool Bioscience	S0197-9
AAV2/9-U6-sgRNA1-sgRNA2(*GLP-1R*)-hSyn-DIO-mCherry	Taitool Bioscience	Custom
AAV2/9-U6-sgRNA(*LacZ*)-hSyn-DIO-mCherry	Taitool Bioscience	Custom
AAV2/9-hSyn-DIO-GLP-1R-3HA-T2A-BFP-WPRE-pA	Taitool Bioscience	Custom

Supplemental Table 2.The ingredient list of standard chow, high-sucrose, and high-fat food.

	Standard Chow		High-Sucrose Food		High-Fat Food	
	gm%	kcal%	gm%	kcal%	gm%	kcal%
Protein	14	15	14	15	26	20
Carbohydrate	73	76	73	76	26	20
Fat	4	9	4	9	35	60
Total		100		100		100
kcal/gm	3.8		3.8		5.2	
**Ingredient**	**gm**	**kcal**	**gm**	**kcal**	**gm**	**kcal**
Casein	140	560	140	560	200	800
L-Cystine	1.8	7.2	1.8	7.2	3	12
Corn Starch	495.692	1982.768	355.7	1422.8	0	0
Maltodextrin 10	125	500	125	500	125	500
Sucrose	100	400	240	960	68.8	275
Cellulose, BW200	50	0	50	0	50	0
Soybean Oil	40	360	40	360	25	225
t-Butylhydroquinone	0.008	0	0.008	0	0	0
Lard	0	0	0	0	245	2205
Mineral Mix S10022M	35	0	35	0	0	0
Mineral Mix S10026	0	0	0	0	10	10
DiCalcium Phosphate	0	0	0	0	13	0
Calcium Carbonate	0	0	0	0	5.5	0
Potassium	0	0	0	0	16.5	0
Vitamin Mix V10037	10	40	10	40	0	0
Vitamin Mix V10001	0	0	0	0	10	40
Choline Bitartrate	2.5	0	2.5	0	2	0
FD&C Yellow Dye #5	0	0	0.05	0	0	0
FD&C Blue Dye #1	0	0	0	0	0.05	0
Total	1000	3850	1000.058	3850	773.85	4057

Supplemental Table 3. The information of antibodies.

Antibodies	Source	Identifier
Anti-c-Fos (9F6) rabbit mAb (1:1,000 immunofluorescence)	Cell Signaling Technology	Catalogue no. 2250, RRID:AB_2247211
Anti-c-Fos (2H2) mouse mAb (1:1,000 immunofluorescence)	Abcam	Catalogue no. ab208942, RRID:AB_2747772
Anti-GLP-1R rabbit mAb (1:500 immunofluorescence)	Abcam	Catalogue no. ab218532, RRID:AB_2864762
Anti-Cre Recombinase mouse mAb (1:500 immunofluorescence)	Merckmillipore	Catalogue no. MAB3120, RRID:AB_2085748
Anti-HA.11 Epitope Tag mouse mAb (1:500 immunofluorescence)	BioLegend	Catalogue no. 901501, RRID:AB_2565006
Anti-GFP rabbit pAb (1:2,000 immunofluorescence)	Abcam	Catalogue no. ab290, RRID:AB_1607841
Anti-GFP chicken pAb (1:2,000 immunofluorescence)	Abcam	Catalogue no. 13970, RRID:AB_300798
Anti-mCherry chicken pAb (1:2,000 immunofluorescence)	Abcam	Catalogue no. ab205402, RRID:AB_2722769
Alexa Fluor 488 goat anti-rabbit (1:500 immunofluorescence)	Thermo Fisher Scientific	Catalogue no. A-11008, RRID:AB_143165
Alexa Fluor 488 goat anti-mouse (1:500 immunofluorescence)	ThermoFisher Scientific	Catalogue no. A-11001, RRID:AB_2534069
Alexa Fluor 488 goat anti-chicken (1:500 immunofluorescence)	Thermo Fisher Scientific	Catalogue no. A-11039, RRID:AB_2534096
Alexa Fluor 555 goat anti-rabbit (1:500 immunofluorescence)	Thermo Fisher Scientific	Catalogue no. A-21428, RRID:AB_2535849
Alexa Fluor 555 goat anti-mouse (1:500 immunofluorescence)	Thermo Fisher Scientific	Catalogue no. A32727, RRID:AB_2633276
Alexa Fluor 555 goat anti-chicken (1:500 immunofluorescence)	Thermo Fisher Scientific	Catalogue no. A-21437, RRID:AB_2535858
Alexa Fluor 647 goat anti-rabbit (1:500 immunofluorescence)	Thermo Fisher Scientific	Catalogue no. A-21245, RRID:AB_141775

Supplemental Table 4. Sample size and sex distribution for figures.

				N (Males)	N （Females)	age
Figure 1	B	GLP-1R Expression		3	-	3 month
		c-Fos Expression	Saline	4	-	3 month
			Liraglutide	4	-	3 month
	E		Saline	1	2	3-4 month
			Liraglutide	1	3	3-4 month
	H			1	1	3 month
Figure 2	B		*sgLacZ*	5	-	4-5 month
			sgGLP-1R	5	-	4-5 month
	C-H		*sgLacZ*	10	-	3-6 month
			sgGLP-1R	11	-	3-6 month
	J		*sgLacZ*	5	2	3-4 month
			sgGLP-1R	6	2	3-4 month
	K		*sgLacZ*	7	-	3-4 month
			sgGLP-1R	11	-	3-4 month
Figure 3	B		EYFP	8	-	3-4 month
			TeNT	9	-	3-4 month
	C		EYFP	9	5	3-4 month
			TeNT	13	4	3-4 month
	D		EYFP	5	3	3-4 month
			TeNT	9	2	3-4 month
	E		EYFP	4	2	3-4 month
			TeNT	5	3	3-4 month
	I	Chow-100 μg/kg	EYFP	2	8	3-4 month
			TeNT	2	7	3-4 month
	J	HSF-100 μg/kg	EYFP	-	5	3-4 month
			TeNT	-	5	3-4 month
	K	Chow-200 μg/kg	EYFP	6	5	3-4 month
			TeNT	6	4	3-4 month
	L	HSF-200 μg/kg	EYFP	4	2	3-4 month
			TeNT	4	2	3-4 month
Figure 4	D-F		GCaMP6	6	4	3-4 month
	G-L		GCaMP6	5	2	3-4 month
	M		GCaMP6	4	2	3-4 month
Figure 5	B		Saline	3	1	4-5 month
			CNO	2	2	4-5 month
	D	Chow	mCherry	7	-	3-4 month
			hM3D	12	-	3-4 month
		HSF	mCherry	7	-	3-4 month

			hM3D	9	3	3-4 month
	E		mCherry	4	4	3-4 month
			hM3D	6	3	3-4 month
	G-H		mCherry	4	1	3-4 month
			hM3D	5	2	3-4 month
	K		ChR2	-	6	4-5 month
S1	C		*GLP-1R* +/+ mice	2	-	2 month
			GLP-1R +/- mice	2	-	2 month
			GLP-1R -/- mice	2	1	2 month
S2	C			5	-	3-4 month
	F		Saline	2	1	4-5 month
			Liraglutide	2	1	4-5 month
	H-K			8	-	3-4 month
S3	A-D		*sgLacZ*	10	-	3-4 month
			sgGLP-1R	11	-	3-4 month
	E		*sgLacZ*	11	-	3-4 month
			sgGLP-1R	10	-	3-4 month
	F-I		*sgLacZ*	10	-	3-6 month
			sgGLP-1R	11	-	3-6 month
S4	C-D		*sgLacZ*	11	-	3-5 month
			sgGLP-1R	15	-	3-5 month
	F-G		*sgLacZ*	4	3	3-4 month
			sgGLP-1R	4	3	3-4 month
S5	C		EYFP	2	3	4-5 month
			GLP-1R OE	4	4	4-5 month
	D-G		EYFP	9	-	3-4 month
			GLP-1R OE	10	-	3-4 month
	H		EYFP	6	-	3-4 month
			GLP-1R OE	9	-	3-4 month
	I-P		EYFP	4	2	3-4 month
			GLP-1R OE	3	3	3-4 month
S7	A		EYFP	6	2	3-4 month
			TeNT	8	2	3-4 month
	B		EYFP	8	6	3-4 month
			TeNT	9	7	3-4 month
	C-E		EYFP	7	-	3-4 month
			TeNT	7	-	3-4 month
	F-M		EYFP	3	4	3-4 month
			TeNT	3	4	3-4 month
	N-O		EYFP	8	4	3-4 month

			TeNT	7	5	3-4 month
	Q-R		EYFP	3	4	3-4 month
			TeNT	3	4	3-4 month
S8	A-B	100 µg/kg	EYFP	2	8	3-4 month
			TeNT	2	7	3-4 month
		200 µg/kg	EYFP	6	5	3-4 month
			TeNT	6	4	3-4 month
	C-D	100 µg/kg	EYFP	-	5	3-4 month
			TeNT	-	5	3-4 month
		200 µg/kg	EYFP	4	2	3-4 month
			TeNT	4	2	3-4 month
S9	A-B		mCherry	7	-	3-4 month
			hM3D	3	3	3-4 month
	E		ChR2	5	3	3-4 month

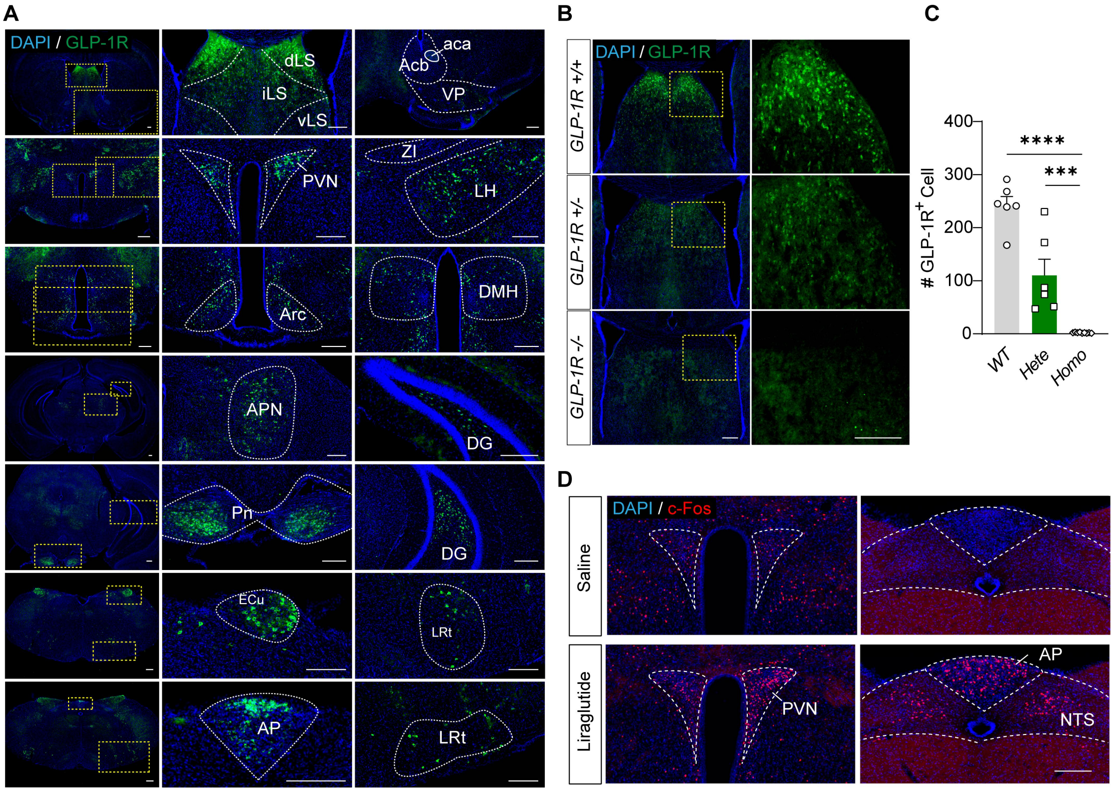

Supplemental Figure 1. Overview of GLP-1R-positive cell distribution, c-Fos expression post-liraglutide systemic administration.

(A) Whole-brain images showcasing the distribution of GLP-1R-positive cells revealed by immunostaining experiments. The scale bar represents 200 µm. (B) Immunofluorescent staining of GLP-1R in wild-type (*GLP-1R +/+*), heterozygous (*GLP-1R +/-*) and homozygote (*GLP-1R -/-*) mice. The scale bar represents 200 µm. (C) Quantification of GLP-1R positive neurons in the LS region in wild-type, heterozygous and homozygote mice. Unpaired two-tail t-test: *WT* vs *Homo* $t_{(13)} = 17.57$, $P < 0.0001$; *Hete* vs *Homo* $t_{(13)} = 4.465$, $P = 0.006$. *** $P < 0.001$ and **** $P < 0.0001$, Means ± s.e.m. (D) Images highlight c-Fos expression in the PVN and hindbrain following injections of either saline or liraglutide. The scale bar indicates 200 µm.

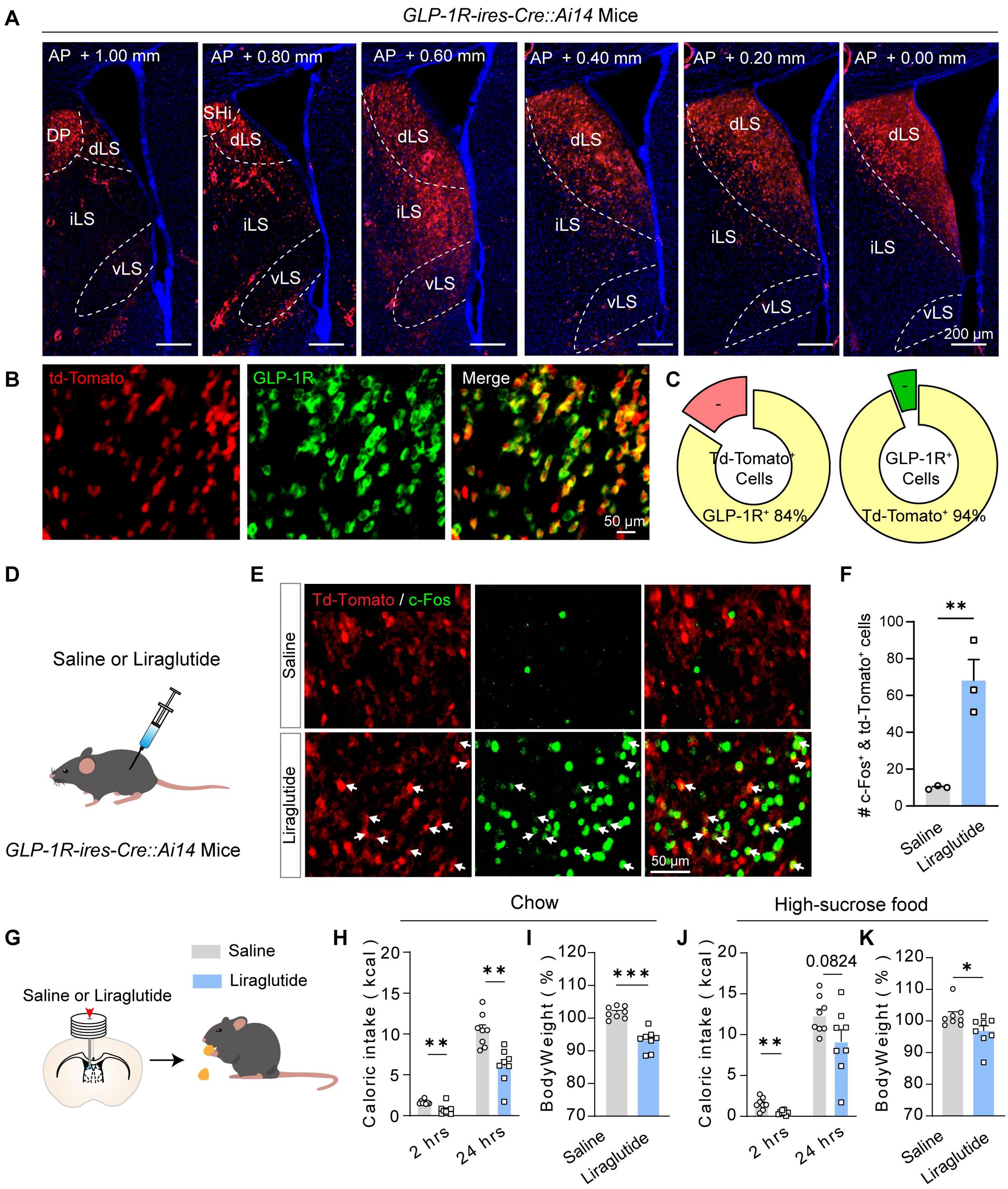

Supplemental Figure 2. The c-Fos expression in LS$^{GLP\text{-}1R}$ neurons following systemic administration of liraglutide, and alterations in food intake and bodyweight due to dorsal LS liraglutide injection.

(A) Images represent Td-Tomato-expressing GLP-1R-positive somatic cells ranging from the rostral to the caudal portion of the lateral septum in *GLP-1R-ires-Cre::Ai14* mice. (B) Representative fluorescence depictions of the dorsal LS showcasing Td-Tomato expression (red) contrasted with immunohistochemistry of GLP-1R (green). (C) The left panel provides quantitative analysis suggesting that the majority of Td-Tomato-expressing neurons in the dorsal LS of *GLP-1R-ires-Cre:: Ai14* mice are GLP-1R positive. The right panel delivers quantitative analysis, indicating that most neurons expressing GLP-1R in the dorsal LS of *GLP-1R-ires-Cre:: Ai14* mice also express Td-Tomato. (D) Experimental schematic illustrating the paradigm for analyzing the level of c-Fos expression after injection of either liraglutide or saline among the

GLP-1R-ires-Cre:: *Ai14* mice. (E) Representative image showing c-Fos expression in $LS^{GLP\text{-}1R}$ neurons induced by liraglutide i.p injection, not saline. (F) Quantification of c-Fos^{+} td-$Tomato^{+}$ cells post administration of saline or liraglutide. Unpaired two-tailed t test. $t_{(4)}=5.023$, P = 0.0074. Means ± s.e.m. (G) Experimental schematic illustrating the paradigm for analyzing food intake and bodyweight changes after dorsal LS injection of either liraglutide or saline. (H-K) Post intra-LS liraglutide injection, a reduction in cumulative caloric intake and bodyweight was observed. Mice were provided with standard chow in figures H-I and high-sucrose food in figures J-K. Paired two-tailed t test. Chow- caloric intake: 2 hrs: $t_{(7)} = 3.768$, P = 0.0070; 24 hrs: $t_{(7)} = 3.923$, P = 0.0057. Chow-bodyweight: $t_{(7)} = 6.497$, P = 0.0003. HSF- caloric intake: 2 hrs: $t_{(7)} = 4.765$, P = 0.0020; 24 hrs: $t_{(7)} = 2.026$, P = 0.0824. HSF - bodyweight: $t_{(7)} = 2.424$, P = 0.0458. Means ± s.e.m.

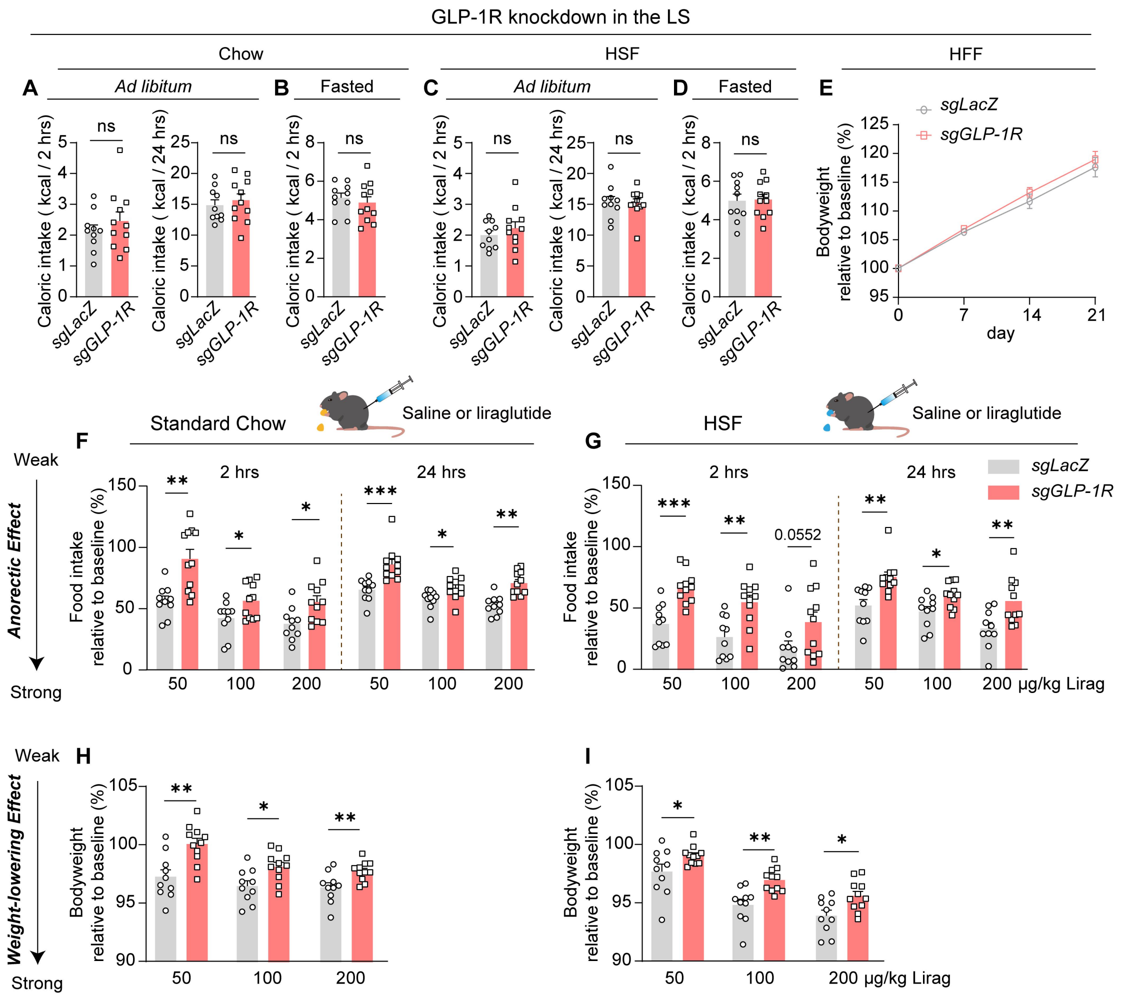

Supplemental Figure 3. Effect of GLP-1 receptor knockdown in LS on baseline feeding, bodyweight, and liraglutide response.

(A-D) Neither sated nor fasted mice on a standard chow or high-sucrose-food diet exhibited altered food intake following GLP-1 receptor knockdown in the dorsal LS (gray: LacZ KD mice, n=10; red: GLP-1R KD mice, n=11). Unpaired two-tailed t test. A left: $t_{(19)} = 0.9038$, P = 0.3774; A right: $t_{(19)} = 0.5625$, P = 0.5803; B: $t_{(19)} = 0.6445$, P = 0.5270; C left: $t_{(19)} = 0.8802$, P = 0.3897; C right: $t_{(19)} = 0.003848$, P = 0.9970; D: $t_{(19)} = 0.1250$, P = 0.9018. Means ± s.e.m. (E) Bodyweight remained unaffected by GLP-1 receptor knockdown in the dorsal LS for mice on a high-fat diet without liraglutide treatment (gray: control mice, n=11; red: GLP-1R knockdown mice, n=10). Two-way repeated-measures ANOVA: interaction: $F_{(3,57)} = 0.4335$, P = 0.7299. virus: $F_{(1,19)} = 0.6543$, P = 0.4286. Means ± s.e.m. (F and G) Attenuation of liraglutide's anorectic effects following GLP-1R knockdown in the dorsal LS on standard chow (F) and a high-sucrose diet (G) for 2 or 24 hours. Statistical results are provided for varying dosages and durations. Unpaired two-tailed test. Standard chow: 50 μg/kg-2 hrs: $t_{(19)} = 3.842$, P = 0.0011; 50 μg/kg-24 hrs: $t_{(19)} = 3.996$, P = 0.0008; 100 μg/kg-2 hrs: $t_{(19)} = 2.269$, P = 0.0351; 100 μg/kg-24 hrs: $t_{(19)} = 2.450$, P = 0.0241; 200 μg/kg-2 hrs: $t_{(19)} = 2.712$, P = 0.0138; 200 μg/kg-24 hrs: $t_{(19)} = 4.822$, P = 0.0001. HSF: 50 μg/kg-2 hrs: $t_{(19)} = 4.626$, P = 0.0002; 50 μg/kg-24 hrs: $t_{(19)} = 3.581$, P = 0.0020; 100 μg/kg-2 hrs: $t_{(19)} = 3.630$, P = 0.0018; 100 μg/kg-24 hrs: $t_{(19)} = 2.692$, P = 0.0144; 200 μg/kg-2 hrs: $t_{(19)} = 2.043$, P = 0.0552; 200 μg/kg-24 hrs: $t_{(19)} = 2.909$, P = 0.0090. Means ± s.e.m. (H and I) Attenuation of the weight-lowering effect of acutely delivered systemic liraglutide following

GLP-1R knockdown in the dorsal LS on standard chow (H) or a high-sucrose diet (I). Standard chow: 50 μg/kg: $t_{(19)} = 3.627$, $P = 0.0018$; 100 μg/kg: $t_{(19)} = 2.805$, $P = 0.0113$; 200 μg/kg: $t_{(19)} = 2.928$, $P = 0.0086$. HSF: 50 μg/kg: $t_{(19)} = 2.105$, $P = 0.048$; 100 μg/kg: $t_{(19)} = 3.804$, $P = 0.0012$; 200 μg/kg: $t_{(19)} = 2.776$, $P = 0.0120$. Means ± s.e.m.

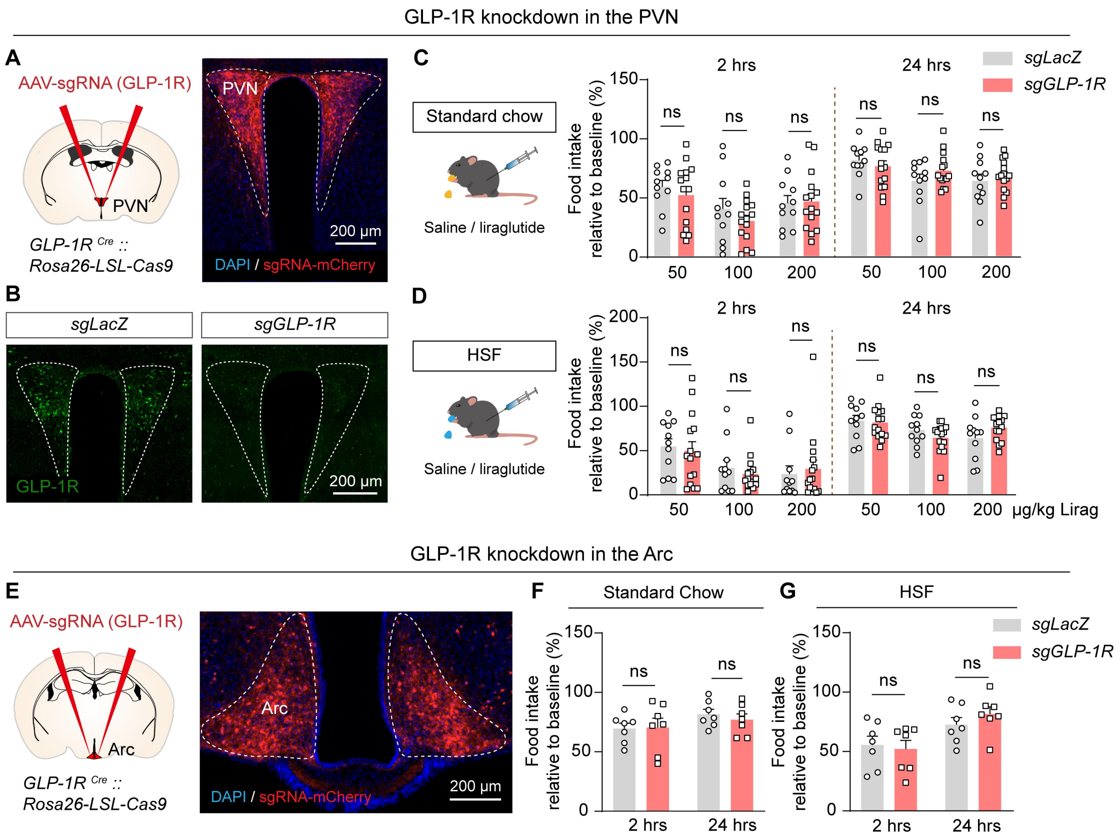

Supplemental Figure 4. Effect of GLP-1 receptor knockdown on liraglutide response across brain regions.

(A-B) An image demonstrates the use of the *sgGLP-1R* virus to target and knock down GLP-1 receptors in the PVN. (C-D) Absence of effect on liraglutide's anorectic response from GLP-1R knockdown in the PVN during standard chow (C) or high-sucrose diet (D) for 2 or 24 hours. Unpaired two-tailed t test. Standard chow: 50 µg/kg-2 hrs: $t_{(24)} = 0.7643$, P = 0.4522; 50 µg/kg-24 hrs: $t_{(24)} = 0.7834$, P = 0.4411; 100 µg/kg-2 hrs: $t_{(24)} = 1.071$, P = 0.2948; 100 µg/kg-24 hrs: $t_{(24)} = 1.315$, P = 0.2010; 200 µg/kg-2 hrs: $t_{(24)} = 0.1666$, P = 0.8690; 200 µg/kg-24 hrs: $t_{(24)} = 0.6417$, P = 0.5271. HSF: 50 µg/kg-2 hrs: $t_{(24)} = 0.6301$, P = 0.7219; 50 µg/kg-24 hrs: $t_{(24)} = 0.2634$, P = 0.7945; 100 µg/kg-2 hrs: $t_{(24)} = 0.6901$, P = 0.4968; 100 µg/kg-24 hrs: $t_{(24)} = 1.282$, P = 0.2121; 200 µg/kg-2 hrs: $t_{(24)} = 0.3966$, P = 0.6952; 200 µg/kg-24 hrs: $t_{(24)} = 1.580$, P = 0.1272. (E) Schematic showing *sgGLP-1R* viral injections and a representative image of viral expression in the Arc. (F-G) Knocking down GLP-1 receptors in the Arc had no noticeable impact on the appetite-reducing effect of acute systemic liraglutide (50 µg/kg) during a standard chow diet (F) or high-sucrose diet (G). Data for standard chow diet are: 2 hrs-$t_{(12)} = 0.9175$, P = 0.1057; 24 hrs-$t_{(12)} = 0.4930$, P = 0.7071. For high-sucrose diet: 2 hrs-$t_{(12)} = 0.3323$, P = 0.7454; 24 hrs-$t_{(12)} = 1.048$, P = 0.3153.

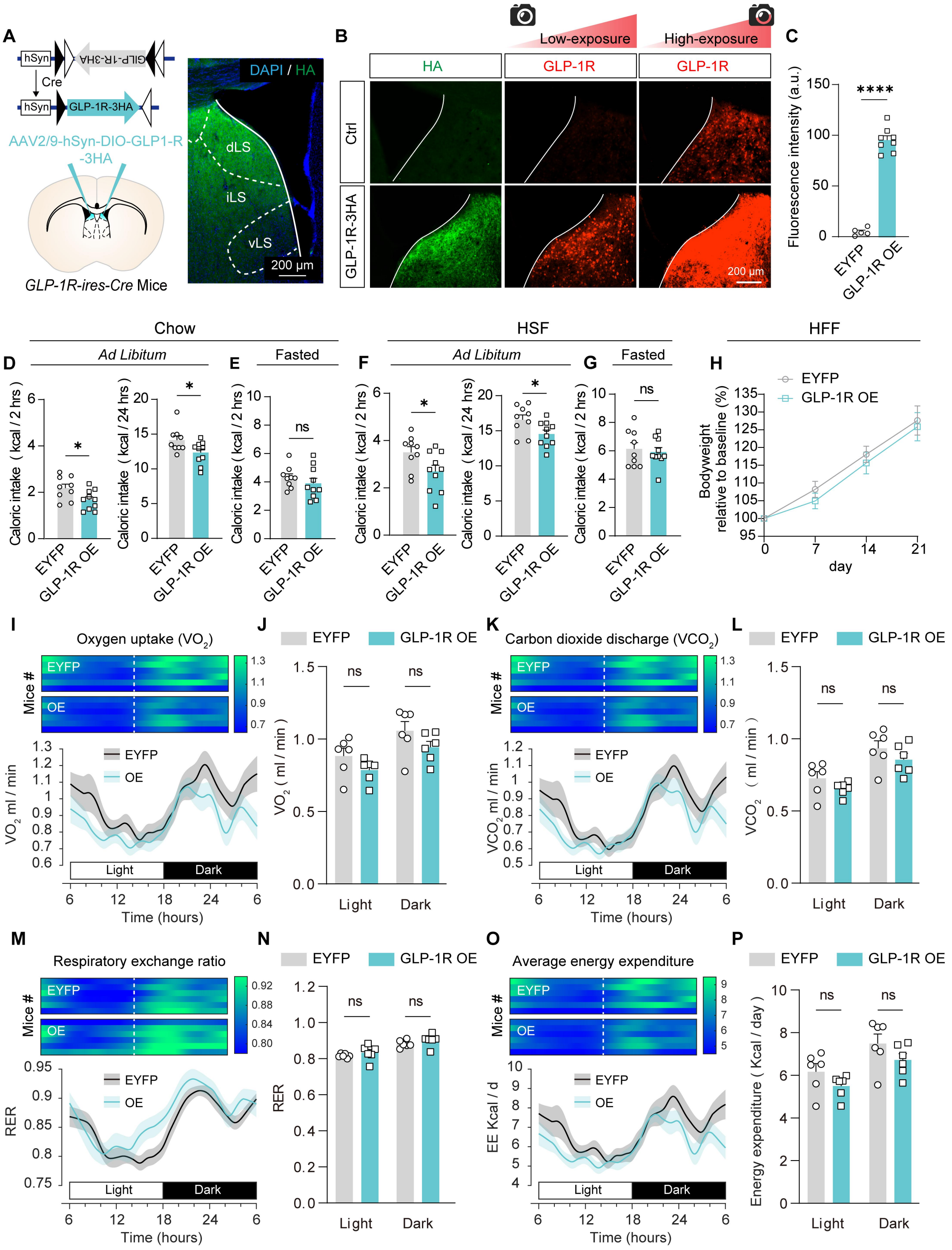

Supplemental Figure 5. Overexpression of GLP-1Rs in the LS reduces food intake in satiated mice without affecting metabolism.

(A) Schematic showing viral injections and representative image of specific expression of GLP-1R-3HA in LS$^{GLP\text{-}1R}$ neurons. (B) Representative image showing overexpression of GLP-1 receptors in dorsal LS. (C) Quantitation of GLP-1R fluorescence intensity in dLS of EYFP- and GLP-1R- mice (gray: EYFP mice, n = 5; blue: GLP-1R mice, n = 8). Unpaired two-tailed t test. $t_{(11)} = 16.09$, $P < 0.0001$. Means ± s.e.m. (D, F) Overexpression of GLP-1 receptors in dorsal LS would decrease the food intake among satiated mice fed with a standard chow (D) or

high-sucrose-food (F) diet. Unpaired two-tailed t test: chow-2 hrs (D, left): $t_{(17)}$ = 2.415, P = 0.0273; chow-24 hrs (D, right):: $t_{(17)}$ = 2.279, P = 0.0358; HSF-2 hrs (F, left): $t_{(17)}$ = 2.238, P = 0.0389; HSF-24 hrs (F, right): $t_{(17)}$ = 2.325, P = 0.0327. Means ± s.e.m. (E, G) Overexpression of GLP-1 receptors in dorsal LS could not affect food consumption among fasted mice fed with a standard chow (E) or high-sucrose-food (G) diet. Chow-2 hrs (E): unpaired two-tailed t test. $t_{(17)}$ = 1.572, P = 0.1344. HSF-2 hrs (G): unpaired two-tailed t test. $t_{(17)}$ = 0.4280, P = 0.6740. Means ± s.e.m. (H) Bodyweight remained unaffected by overexpression of GLP-1 receptors in dorsal LS for mice on a high-fat diet (gray: control mice, n=6; blue: GLP-1R OE mice, n=9). Two-way repeated-measures ANOVA: interaction: $F_{(3, 39)}$ = 0.2505, P = 0.8605. virus: $F_{(1, 13)}$= 0.3455, P = 0.5667. Means ± s.e.m. (I-J) Oxygen uptake of EYFP- and GLP-1R OE mice during 24 hrs. Two-way repeated-measures ANOVA: $F_{(1, 10)}$ = 2.399, P = 0.1525. Means ± s.e.m. (K-L) Carbon dioxide discharge of EYFP- and GLP-1R OE mice during 24 hrs. Two-way repeated-measures ANOVA: $F_{(1, 10)}$ = 1.766, P = 0.2135. Means ± s.e.m. (M-N) Respiratory exchange ratio of EYFP- and GLP-1R OE mice during 24 hrs. Two-way repeated-measures ANOVA: $F_{(1, 10)}$ = 1.409, P = 0.2627. Means ± s.e.m. (O-P) Energy expenditure of EYFP- and GLP-1R OE mice during 24 hrs. Two-way repeated-measures ANOVA: $F_{(1, 10)}$ = 2.305, P = 0.1599. Means ± s.e.m.

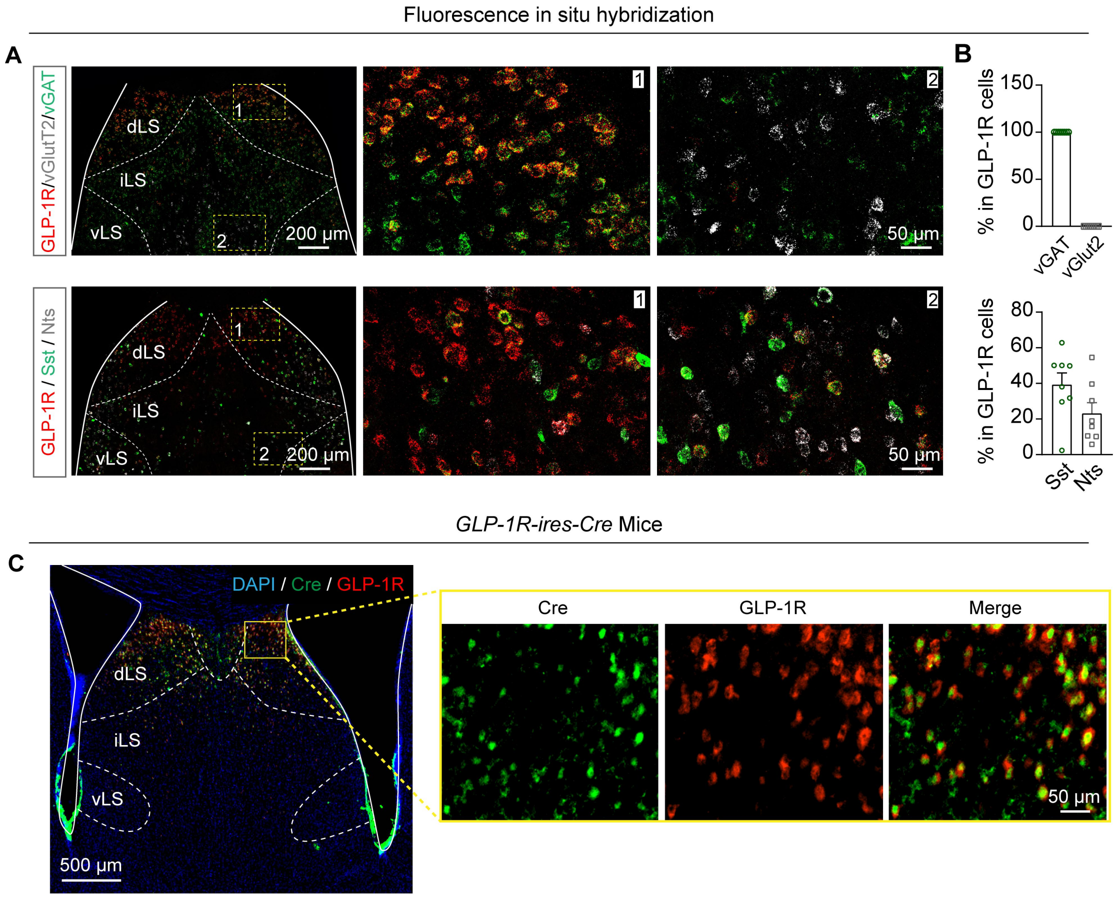

Supplemental Figure 6. Validation of *GLP-1R-ires-Cre* mice and co-localization analysis of LS$^{GLP\text{-}1R}$ neurons with other biomarkers.

(A) *In situ* hybridization schematic depicting the co-localization of GLP-1R with vGAT and vGlut2, and GLP-1R with Sst and Nts in the LS region. (B) Statistical analysis of GLP-1R co-localization with vGAT, vGlut2, Sst, and Nts in the LS region. (C) Displayed are representative fluorescence images of the dorsal LS, detailing immunohistochemistry for Cre (green) and GLP-1R (red).

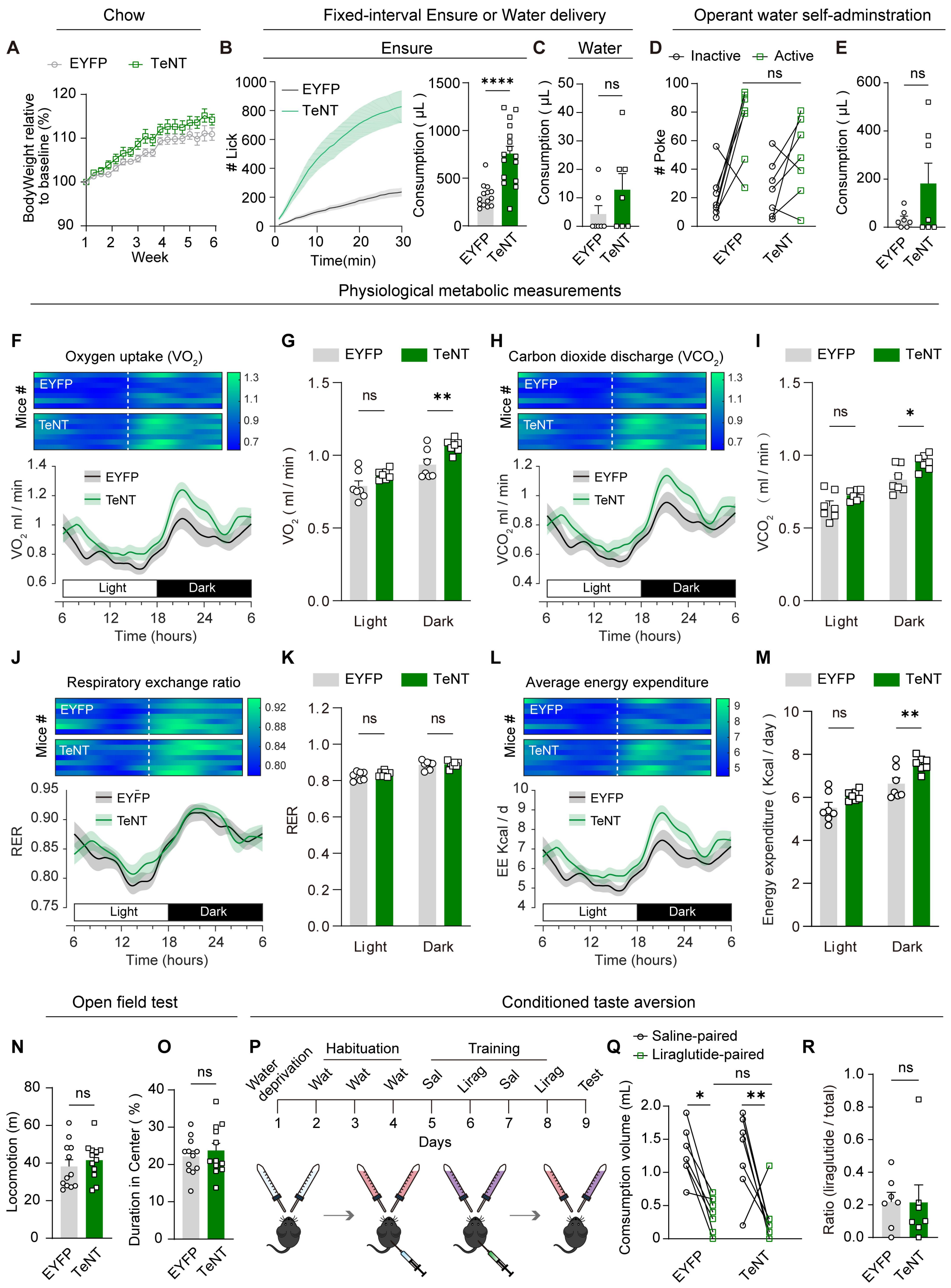

Supplemental Figure 7. The effects of silencing LS^{GLP-1R} neurons on the intake of Ensure and water, as well as on metabolism, anxiety levels, and liraglutide-induced nausea.

(A) Bodyweight gain quantification following EYFP- and TeNT-expressing fed on standard chow. Two-way repeated-measures ANOVA: $F_{(1, 16)} = 3.641$, $P = 0.0745$. Means ± s.e.m. (B) Synaptic silencing of LS^{GLP-1R} neurons increased the number of licks to the spout and Ensure solution consumption during the fixed-interval food delivery paradigm (gray: EYFP mice, n = 14; green: TeNT mice, n = 16). Unpaired two-tailed t test. $t_{(28)} = 5.044$, $P < 0.0001$. Means ± s.e.m. (C)

Synaptic silencing of LS^{GLP-1R} neurons would not affect water consumption during the free consumption paradigm (gray: EYFP mice, n = 7; green: TeNT mice, n = 7). Unpaired two-tailed t test. $t_{(12)} = 1.342$, P = 0.2046. Means ± s.e.m. (D-E) Synaptic silencing of LS^{GLP-1R} neurons would not affect the number of pokes at active ports (D) and water consumption (E) during the poke-based water intake paradigm. D: Two-way repeated-measures ANOVA: $F_{(1, 12)} = 15.29$, P = 0.0021, followed by Sidak's post hoc test. E: Unpaired two-tailed t test. $t_{(12)} = 1.468$, P = 0.1679. Means ± s.e.m. (F-G) Oxygen uptake of EYFP- and TeNT-expressing mice during 24 hrs. Two-way repeated-measures ANOVA: $F_{(1, 12)} = 7.364$, P = 0.0188, followed by Sidak's post hoc test. **P < 0.01. Means ± s.e.m. (H-I) Carbon dioxide discharge of EYFP- and TeNT-expressing mice during 24 hrs. Two-way repeated-measures ANOVA: $F_{(1, 12)} = 7.117$, P = 0.0205, followed by Sidak's post hoc test. *P < 0.05. Means ± s.e.m. (J-K) Respiratory exchange ratio of EYFP- and TeNT- expressing mice during 24 hrs. Two-way repeated-measures ANOVA: $F_{(1, 12)} = 0.6876$, P = 0.4232. Means ± s.e.m. (L-M) Energy expenditure of EYFP- and TeNT-expressing mice during 24 hrs. Two-way repeated-measures ANOVA: $F_{(1, 12)} = 7.482$, P = 0.0181, followed by Sidak's post hoc test. **P < 0.01. Means ± s.e.m. (N-O) Synaptic silencing of LS^{GLP-1R} neurons would not affect the locomotion (N) and time in the center (O) during the open field test (gray: EYFP mice, n = 12; green: TeNT mice, n = 12). Locomotion: unpaired two-tailed t test. $t_{(22)} = 0.7121$, P = 0.4839. Duration in the center: unpaired two-tailed t test. $t_{(22)} = 0.6560$, P = 0.5186. Means ± s.e.m. (P) Scheme depicting the conditioned taste aversion (CTA) paradigm. (Q-R) Synaptic silencing of LS^{GLP-1R} neurons would not blunt liraglutide-induced CTA. Q: Two-way repeated-measures ANOVA: $F_{(1, 12)} = 23.48$, P = 0.0004, followed by Sidak's post hoc test. * P < 0.05, **P < 0.01. R: Unpaired two-tailed t test. $t_{(12)} = 0.03025$, P = 0.9764. Means ± s.e.m.

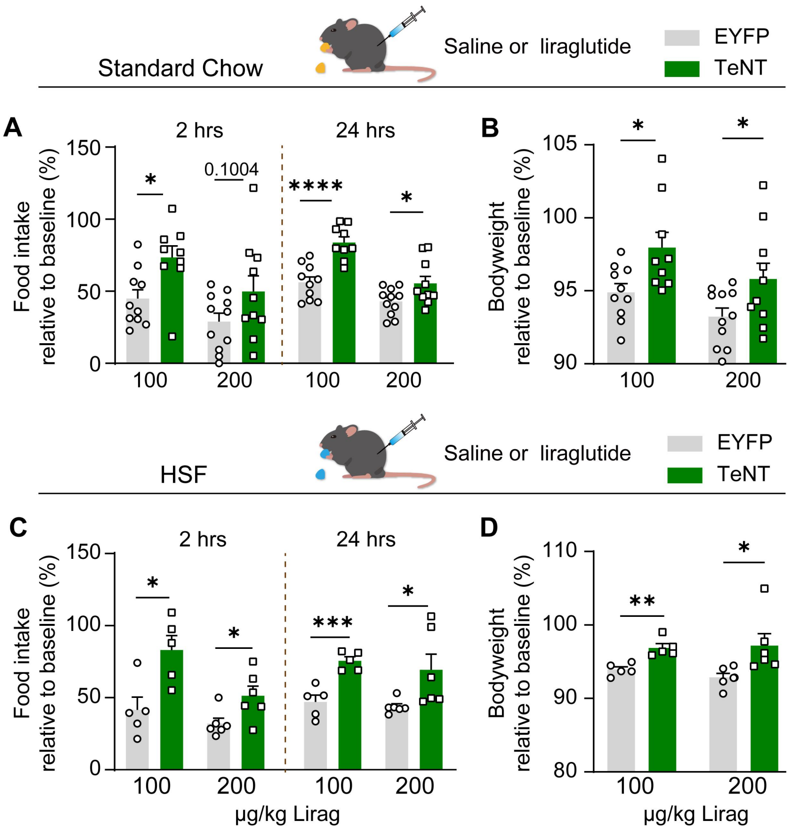

Supplemental Figure 8. Silencing of LS$^{GLP\text{-}1R}$ neurons reduces liraglutide's effects on food intake and bodyweight.

(A and C) Silencing of LS$^{GLP\text{-}1R}$ neurons attenuated the anorectic effect of acutely delivered systemic liraglutide during standard chow (A) or high-sucrose diet (C) over varying durations and dosages. Unpaired two-tailed test. Standard chow: 100 μg/kg-2 hrs: $t_{(17)} = 2.835$, $P = 0.0114$; 100 μg/kg-24 hrs: $t_{(19)} = 1.727$, $P = 0.1004$; 200 μg/kg-2 hrs: $t_{(17)} = 5.075$, $P < 0.0001$; 200 μg/kg-24 hrs: $t_{(19)} = 2.252$, $P = 0.0363$. HSF: 100 μg/kg-2 hrs: $t_{(8)} = 3.120$, $P = 0.0142$; 100 μg/kg-24 hrs: $t_{(10)} = 2.479$, $P = 0.0326$; 200 μg/kg-2 hrs: $t_{(8)} = 5.078$, $P = 0.0010$; 200 μg/kg-24 hrs: $t_{(10)} = 2.294$, $P = 0.0447$. Means ± s.e.m. (C and D) Attenuation of the weight-lowering effect of systemic liraglutide by synaptic silencing of LS$^{GLP\text{-}1R}$ neurons during standard chow (C) or high-sucrose diet (D). Unpaired two-tailed test. Standard chow: 100 μg/kg: $t_{(17)} = 2.603$, $P = 0.0186$; 200 μg/kg: $t_{(19)} = 2.173$, $P = 0.0426$. HSF: 100 μg/kg: $t_{(8)} = 4.373$, $P = 0.0024$; 200 μg/kg: $t_{(10)} = 2.516$, $P = 0.0306$. Means ± s.e.m.

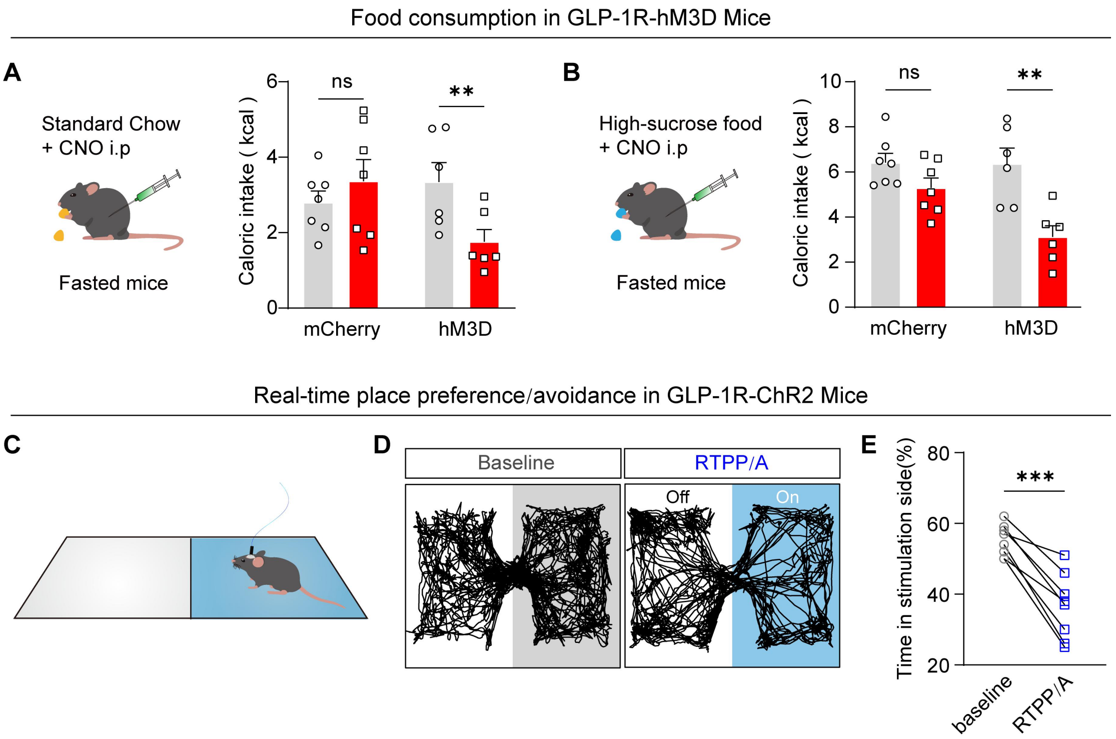

Supplemental Figure 9. Activation of LS$^{GLP\text{-}1R}$ neurons influences caloric intake and aversive behaviors.

(A) CNO injection reduced standard chow food intake in LS$^{GLP\text{-}1R}$-hM3D-expressing (n=6 animals) but not mCherry-expressing fasted mice (n=7 animals). Two-way repeated-measures ANOVA, $F_{(1, 11)} = 17.18$, $P = 0.0016$, followed by Sidak's post hoc test. $^{**}P < 0.01$. Means ± s.e.m. (B) CNO injection reduced high-sucrose food intake in LS$^{GLP\text{-}1R}$-hM3D-expressing (n = 6 animals) but not mCherry-expressing fasted mice (n=7 animals). Two-way repeated-measures ANOVA, $F_{(1, 11)} = 5.212$, $P = 0.0433$, followed by Sidak's post hoc test. $^{**}P < 0.01$. Means ± s.e.m. (C) Scheme depicting the real-time place preference/avoidance (RTPP/A) paradigm. (D) Representative locomotor trace of an LS$^{GLP\text{-}1R}$::ChR2 mouse that received 20-Hz photostimulation in the 'Laser' compartment. (E) LS$^{GLP\text{-}1R}$::ChR2 mice spent less time in the photostimulated side of the RTPP chamber. Paired two-tailed t test. $t_{(7)} = 7.327$, $P = 0.0002$.

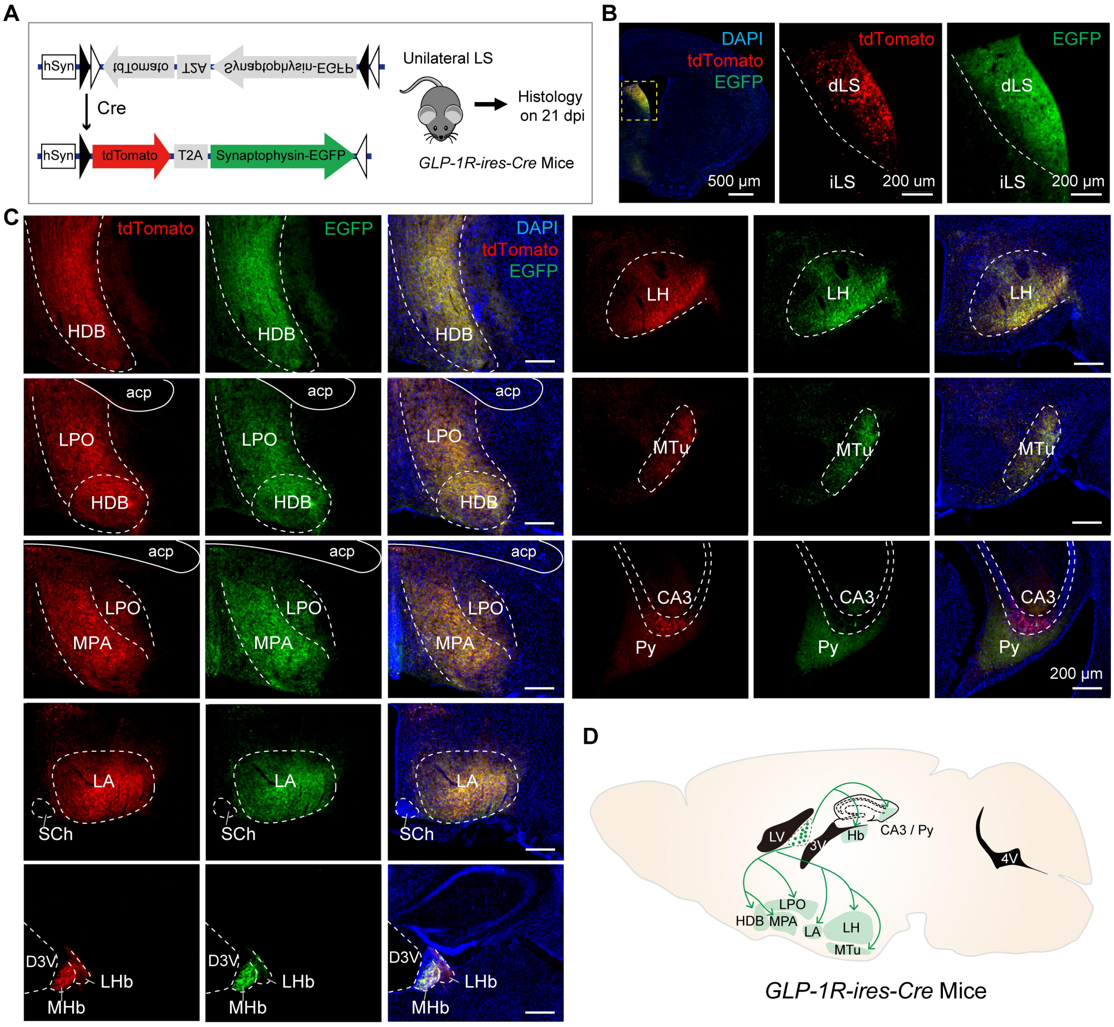

Supplemental Figure 10. Mapping the projections of LS$^{GLP\text{-}1R}$ neurons.

(A) Schematic showing the SynaptoTag AAV strategy to map the projections of LS$^{GLP\text{-}1R}$ neurons. (B) Representative image of the injection site and viral expression in the LS of *GLP-1R-ires-Cre* mice. (C) Representative image showing tdTomato-expressing axons and GFP-expressing axon terminals in different regions. (D) To culminate, a schematic consolidates the information into a comprehensive projection map, depicting the expansive reach of LS$^{GLP\text{-}1R}$ neurons across the brain.

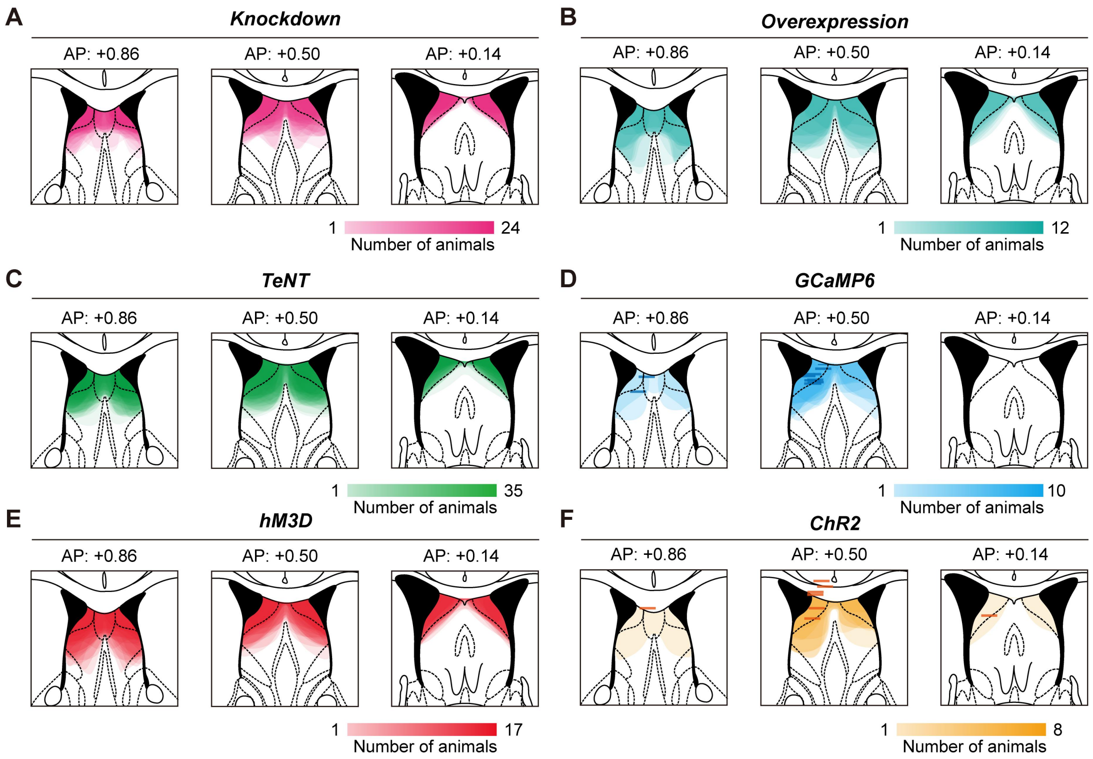

Supplemental Figure 11. Locations of virus expression and optic fiber placement.

(A) Schematics illustrating *sgGLP-1R* virus expression in the LS of *GLP-1R-ires-Cre:: LSL-Cas9* mice, as related to the experiments shown in Figure 2 and Supplemental Figure 3. (B) Schematics illustrating GLP-1R-3HA virus expression in the LS of *GLP-1R-ires-Cre* mice, as related to the experiments shown in Supplemental Figure 5. (C) Schematics illustrating TeNT-2A-EGFP virus expression in the LS of *GLP-1R-ires-Cre* mice, as related to the experiments shown in Figure 3, Supplemental Figure 7 and Supplemental Figure 8. (D) Schematics illustrating GCaMP virus expression and optic fiber locations in the LS of *GLP-1R-ires-Cre* mice, as related to the experiments shown in Figure 4. (E) Schematics illustrating hM3D-mCherry virus expression in the LS of *GLP-1R-ires-Cre* mice, as related to the experiments shown in Figure 5, A-H and Supplemental Figure 9, A and B. (F) Schematics illustrating ChR2-mCherry virus expression and optic fiber locations in the LS of *GLP-1R-ires-Cre* mice, as related to the experiments shown in Figure 5, I-K and Supplemental Figure 9, C-E.
